# Nanopore adaptive sampling accurately detects nucleotide variants and improves the characterization of large‐scale rearrangement for the diagnosis of cancer predisposition

**DOI:** 10.1002/ctm2.70138

**Published:** 2025-01-09

**Authors:** Sandy Chevrier, Corentin Richard, Marie Mille, Denis Bertrand, Romain Boidot

**Affiliations:** ^1^ Unit of Molecular Biology Georges‐François Leclerc Cancer center UNICANCER Dijon France; ^2^ SeqOne Genomics Montpellier France

**Keywords:** adaptive sampling, germline variants, large‐scale rearrangements, single nucleotide variation

## Abstract

**Background:**

Molecular diagnosis has become highly significant for patient management in oncology.

**Methods:**

Here, 30 well‐characterized clinical germline samples were studied with adaptive sampling to enrich the full sequence of 152 cancer predisposition genes. Sequencing was performed on Oxford Nanopore (ONT) R10.4.1 MinION flowcells with the Q20+ chemistry.

**Results:**

In our cohort, 11 samples had large‐scale rearrangements (LSR), which were all detected with ONT sequencing. In addition to perfectly detecting the locus of the LSR, we found a known MLPA amplification of exon 13 in the *BRCA1* (NM_7294) gene corresponded to a duplication in tandem of both exons 12 and 13 of the reference NM_7300. Similarly, in another sample with a known total deletion of the *BRCA1* gene, ONT sequencing highlighted this complete deletion was the consequence of a large deletion of almost 140 000 bp carrying over five different genes. ONT sequencing was also able to detect all pathogenic nucleotide variants present in 16 samples at low coverage. As we analyzed complete genes and more genes than with short‐read sequencing, we detected novel unknown variants. We randomly selected six new variants with a coverage larger than 10× and an average quality higher than 14, and confirmed all of them by Sanger sequencing, suggesting that variants detected with ONT (coverage >10× and quality score >14) could be considered as real variants.

**Conclusions:**

We showed that ONT adaptive sampling sequencing is suitable for the analysis of germline alterations, improves characterization of LSR, and detects single nucleotide variations even at low coverage.

**Key points:**

- Adaptive sampling is suitable for the analysis of germline alterations.
- Improves the characterization of Large Scale Rearrangement and detects SNV at a minimum coverage of 10x.
- Allows flexibility of sequencing.

## Graphical abstract

2. The protocol we set up can be immediately used in routine molecular diagnosis labs for the characterization of large‐scale rearrangements.

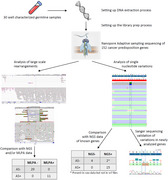

## 1 INTRODUCTION

The third version of sequencing generation has the potential to improve molecular diagnosis by giving a more comprehensive view of the genome. Indeed, with real‐time sequencing, Oxford Nanopore Technologies (ONT) revolutionizes sequencing data generation with the sequencing of native DNA without any PCR amplification or chemical modification.^1^ Moreover, the real‐time sequencing capability allows the specific DNA sequence selection while sequencing, without requiring previous selection during library preparation.^2^ This is called adaptive sampling. During sequencing of long fragments (>6000 bp), single‐strand DNA molecules squeeze through protein nanopores at a speed of 400 nucleotides/second. While sequencing the first 400 nucleotides, an algorithm aligns the sequence on a reference genome and detects whether the DNA sequence corresponds to chromosomal coordinates indicated on a reference file. When the strand sequence fits with the file, the strand is fully sequenced. When the DNA strand does not correspond to the file, the sequencing stops and the DNA strand is rejected. Thus, an enrichment of specific regions is performed in real‐time. Additionally, due to the sequencing data from the non‐selected regions (rejected reads), a low‐pass whole genome sequencing could be obtained.

For years, germline molecular diagnosis has been made increasingly accessible, and large gene panels are now routinely used in clinical practice.^3^ The progress of research and its application in diagnosis requires high responsiveness from routine molecular diagnosis labs. With short‐read sequencing, the selection of genes is performed during library preparation by using capture probes or specific primers in multiplexed PCR. The addition of a new target needs to change the design of probe sets and different validation steps to validate the good selection of all targets. These different steps take time and have a significant cost. With adaptive sampling, an immediate responsiveness is possible and the analysis of new targets can be immediately applied to routine diagnosis. Moreover, long‐read sequencing improves the detection of structural variations.^4^, ^5^, ^6^

In this proof‐of‐concept work, we applied ONT adaptive sampling sequencing to enrich the whole sequence of 152 cancer predisposition genes to assess its capability to detect single nucleotide variations (SNV) and large‐scale rearrangements (LSR) on 30 clinical samples.

## 2 METHODS

### 2.1 Patients and samples

The study was designed as a proof‐of‐concept study by choosing a range of alterations that are observed in a routine clinical diagnosis laboratory (Figure S1). So, we conducted it on germline blood samples from 30 patients (Table 1) diagnosed at the Georges– Francois Leclerc Cancer Center (CGFL, Dijon, France) between 2017 and 2022. These samples have representative alterations we can observe in a routine activity with techniques used in a routine molecular diagnosis lab. All germline samples were sent to our lab for analyses as part of the routine clinical diagnosis procedure for predisposing syndrome to breast, ovary, prostate, pancreas, or digestive tract cancer. All patients gave their consent to use their samples for research after their use for molecular diagnosis. The study was conducted in accordance with the Declaration of Helsinki and approved (approval no. 00010311) by the Ethics Committee of the Georges‑Francois Leclerc Cancer Center (Dijon, France) and by the Consultative Committee of Burgundy (Dijon, France) for the Protection of Persons Participating in Biomedical Research (Comité Consultatif de Protection des Personnes en Recherche Biomédicale de Bourgogne). Written informed consent was provided by all patients.

**TABLE 1:** Patient characteristics.

Samples	Gene	Mutations observed Nucleotide variation protein variation		Classification	Clinical outcome
#1	*BRCA1*	c.5266dup	p.(Gln1756ProfsTer74)	Pathogenic	TNBC at 43 yo
#2	*BRCA1*	c.(4675+1_4676‐1)_(5467+1_5468‐1)del	p.?	Pathogenic	Ovary cancer at 49 yo
#3	*MLH1*	c.1852_1854delAAG	p.(Lys618del)	Pathogenic	MSI‐high colon cancer at 54 yo
#4	*BRCA1*	c.(134+1_135‐1)_(441+1_442‐1)del	p.?	Pathogenic	Ovary cancer at 68 yo
#5	*BRCA1*	c.(670+1_671‐1)_(4185+1_4186‐1)del	p.?	Pathogenic	Ovary cancer at 64 yo
#6	*PALB2*	c.2835‐1G > C	*Splicing*	Pathogenic	BC at 49 yo
#7	*BRCA1*	c.(?_‐232)_(80+1_81‐1)del	p.?	Pathogenic	TNBC at 36 yo
#8	*BRCA1*	c.(?_‐30)_(*220_?)	p.?	Pathogenic	TNBC at 33 yo
#9	*BRCA1*	c.4185+1_4186‐1)_(4357+1_4358‐1)dup	p.?	Pathogenic	TNBC at 36 yo
#10	*BRCA1*	c.4185+1_4186‐1)_(4357+1_4358‐1)dup	p.?	Pathogenic	TNBC at 37 yo
#11	*BRCA2*	c.2376C > A	p.(Tyr792Ter)	Pathogenic	TNBC at 49 yo
#12	*BRCA1*	c.5044_5048delinsT	p.(Glu1682Ter)	Pathogenic	TNBC at 57 yo
#13	*BRCA2*	c.5645C > A	p.(Ser1882Ter)	Pathogenic	Ovary cancer at 46 yo
#14	*BRCA1*	c.(5406+1_5407‐1)_(5467+1_5468‐1)del	p.?	Pathogenic	HR+HER2‐ BC at 49 yo
#15	*No alteration observed*				TNBC at 45 yo
#16	*BRCA1*	c.1116G > A	p.(Trp372Ter)	Pathogenic	TNBC at 34 yo
#17	*MLH1*	c.(306+1_307‐1)_(453+1_454‐1)del	p.?	Pathogenic	Colon cancer at 63 yo
#18	*BRCA2*	c.8586_8589delinsTTCACTAAAAG	p.(Glu2863SerfsTer8)	Pathogenic	HER2+ BC at 38yo
#19	*MLH1*	c.2059C > T	p.(Arg687Trp)	Pathogenic	Prostate cancer at 57 yo
#20	*BRCA1*	c.5154G > A	p.(Trp1718Ter)	Pathogenic	Pancreas cancer at 73 yo
#21	*MLH1*	c.1178T > C	p.(Leu393Pro)	Pathogenic	HR+ BC at 49 yo
#22	*BRCA2*	c.(316+1_317‐1)_(425+1_426‐1)del	p.?	Pathogenic	Unaffected woman whose sister had a BC at 41 yo
#23	*MLH1*	c.2262del	p.(Arg755GlyfsTer28)	Unknown	Colon cancer at 33 yo
#24	*BRCA1* *PALB2*	c.1016dup c.529A > T	p.(Val340GlyfsTer6) p.(Lys177Ter)	Pathogenic Pathogenic	Pancreas cancer at 74 yo
#25	*PALB2*	c.1438A > T	p.(Lys480Ter)	Pathogenic	Bilateral TNBC at 38 yo
#26	*BRCA2*	c.(8487+1_8488‐1)_(8632+1_8633‐1)del	p.?	Pathogenic	TNBC at 33 yo
#27	*BRCA2*	c.5065_5066del	p.(Ala1689LysfsTer5)	Pathogenic	TNBC at 50 yo
#28	No alteration observed				TNBC at 64 yo
#29	No alteration observed				HER2+ BC at 68 yo
#30	No alteration observed				RH+HER2‐ BC at 59 yo

Abbreviations: BC, breast cancer; HR, hormonal receptor; TNBC, triple‐negative breast cancer; yo, years old.

### 2.2 DNA extraction

Three hundred microliters of whole blood were extracted with the Monarch Genomic DNA Purification Kit (New England Biolabs) by following the manufacturer's protocol. The quantity of the extracted genomic DNA was assessed with a fluorimetric method using a Qubit device (Fisher Scientific), and integrity was checked with a Tapestation 4200 device (Agilent Biotechnologies). The size of the DNA obtained was higher than 10 000 bp.

### 2.3 Library preparation

1.2 µg of gDNA extracted from white blood cells was slightly fragmented with g‐TUBE (Covaris) by two spins of 1 min at 6010*g*. Then, libraries were prepared with the LSK114 kit (ONT) following the manufacturer's instructions.

### 2.4 Flow cell loading and sequencing set‐up

R10.4.1 MinION flow cells were prepared following the manufacturer's instructions. When the final library amount was superior to 300 ng (15 µL at 20 ng/µL), half of the library was loaded. The second one was loaded 24 h later after a nuclease wash of the flow cell following the manufacturer's instructions. When the library yield is below 300 ng, the whole library is loaded at once. A bed file containing the chromosomic coordinates (±10 kb upstream and downstream of every complete gene) of 152 cancer‐predisposing genes (Table S1) was uploaded to the MinKnow software, representing a size of 13 964 404 nucleotides corresponding to 0.47% of the human genome. The device used was a GridION containing a GPU Nvidia Quadro GV100.

### 2.5 Bioinformatics analysis

Sequenced reads were mapped using minimap2 (v2.24‐r1122, ‐ax map‐ont –MD)^7^ to the reference genome hg19. From the mapping, SNVs and indels of the 152 predisposing genes were identified using Clair3 (v0.1‐r12, –platform = “ont” –model_path = r1041_e82_400bps_hac_g632 –enable_phasing)^8^ and decomposed/normalized with vt (v0.57721).^9^ Variants were then annotated with the following resources: VEP dbNSFP (v4.3a)^10^ and VEP dbscSNV (v1.1), ClinVar (2023‐01),^11^ gnomAD (v2.1),^12^ OMIM (2021‐12).^13^ Based on the annotations and ACMG guidelines,^14^ variants were automatically classified into five classes: pathogenic, likely pathogenic, uncertain significance, likely benign, and benign.

From the mapping, LSR were detected using Sniffles2 (v2.0.7, –phase –minsvlen 45 –long‐del‐coverage 10 –long‐dup‐coverage 0 –no‐consensus).^15^ Variants were then annotated with the following resources: OMIM (2021‐12), DGV (2016‐03),^16^ MedGen (2022‐10),^17^ ClinGen region and gene (2020‐03),^18^ DECIPHER (HI_Predictions_Version3),^19^ and gnomAD pop frequency and pliscore (v2.1_sv.sites).^20^ Based on the annotations and ACMG guidelines,^21^ LSR were automatically classified in the 5 ACMG classes described above.

### 2.6 Quality score calculation

The quality score, also named the Phred score, is used to define the basecalling quality. This score is an estimated error probability. The quality score is calculated as −10log(E) where E is the estimated error probability. For example, a quality score of 20 corresponds to an error of 1 in 100.

## 3 RESULTS

### 3.1 Technical performances of adaptive sampling sequencing

**FIGURE 1:**
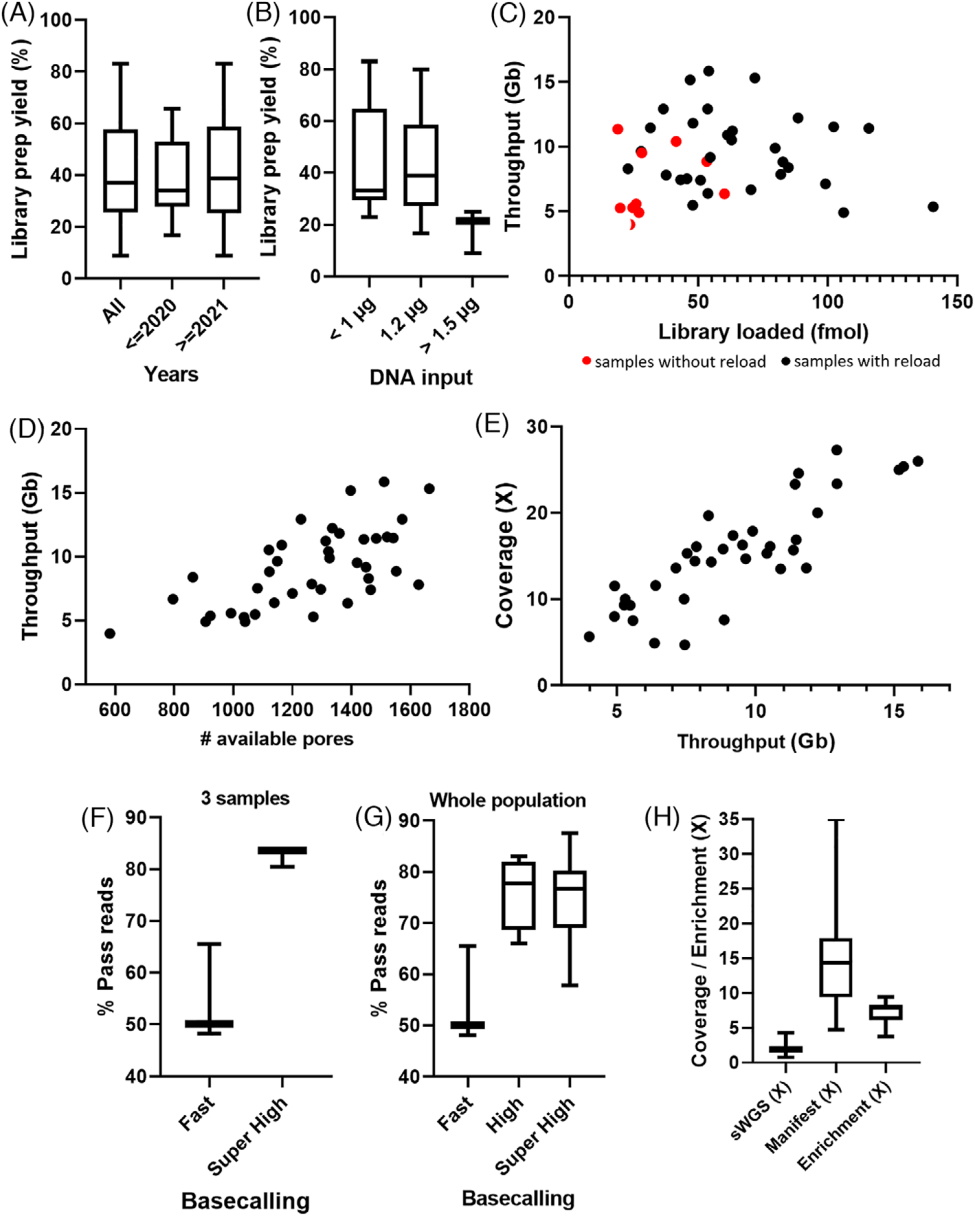
Technical set‐up and performances of ONT adaptive sampling. (A) Library yield is not affected by the age of gDNA extracted from whole blood. The use of gDNA from samples stored before 2020 or from 2021 gives the same library preparation yield. (B) The best yield is obtained when 1.2 µg of gDNA is used for library preparation. While an amount below 1 µg of gDNA resulted in a slight decrease in library preparation yield, a DNA amount higher than 1.5 µg resulted in a dramatic decrease in the library preparation yield. (C) The increase of gDNA amount calculated in fmoles loaded on flow cells tended to improve the sequencing throughput up to 100 fmoles. Higher quantities seemed to be detrimental to sequencing throughput. Moreover, the reload of the flow cells 24 h after the first load seemed to improve the sequencing throughput (black dots). Indeed, samples with no reload (red dots) tended to have a lower sequencing throughput. (D) The number of available pores at the beginning of the sequencing run directly influenced sequencing throughput: the more pores available, the higher the throughput. (E) The amount of Giga bases (Gb) generated throughout the sequencing run was positively correlated with the coverage of target genes. (F) The accuracy of basecalling had a strong influence on the percentage of reads passing quality filters. From the same fast5 files from three samples, whereas only 50% of reads passed quality filters with fast accuracy basecalling, more than 80% of reads passed filters with super‐high accuracy basecalling. (G) Among the three different available basecalling accuracies, high‐ and super‐high accuracies gave similar quality with a mean percentage of pass reads of more than 75% on the whole population. (H) The coverage obtained for the sequences in the manifest was clearly higher than the coverage of the shallow Whole Genome Sequencing obtained from the rejected reads. The enrichment was calculated by dividing the coverage on the manifest by the coverage of the rejected reads. ONT, Oxford Nanopore Technologies.

We first analyzed some technical parameters to provide the best overview of this new chemistry. The first step was the library preparation yield. Indeed, even if the starting material was germline genomic DNA, some samples had been stored at −20°C for several years (mean = 1.5 years, min < 1 year, max = 5 years). The median library preparation yield was 36.98% (quantity of libraries obtained in relation to the initial quantity of gDNA). This yield was not different for samples stored for more than 2 years (median = 34.06%) or less than 2 years (38.77%; Figure 1A). Then, we tested the library preparation yield in relation to the initial quantity of gDNA. When less than 1 µg (min: 525 ng, max: 970 ng) of gDNA is used, the yield was not different from an initial quantity of 1.2 µg recommended by the provider (median = 33.09% and 38.77%, respectively). However, a higher quantity of gDNA (1.5 µg) was linked to a decrease in the library preparation yield (median = 21.35%; Figure 1B). Based on these observations, we can conclude the age of the samples did not influence the yield of the library preparation, and the optimal amount of gDNA to initiate is 1.2 µg, with an average yield of 40.86% (min: 8.86%, max: 83.08%). Consequently, even if we used the same starting amount of gDNA, we were not able to load the same amount of final library on flow cells. We therefore analyzed the sequencing throughput in relation to the amount of library loaded. The increase of the amount in fmoles but not in ng loaded on flow cells tended to improve the sequencing throughput as well as the reload of the flow cells 24 h after the first load (Figure 1C; Figure S2A). Adding up to 100 fmoles is beneficial for throughput, but more than 100 fmoles decreases throughput. Then, we correlated the number of available pores before each run with the sequencing throughput. The number of available pores at the beginning of sequencing is directly related to the sequencing throughput (Figure 1D). Finally, we also observed target gene coverage increases with the throughput (Figure 1E).

One of the specifics of ONT sequencing is the basecalling performed throughout sequencing. Three levels of basecalling accuracy were available: fast accuracy, high accuracy, and super‐high accuracy. As adaptive sampling needs high informatics resources, the super‐high accuracy basecalling cannot be performed simultaneously. We first tested fast‐accuracy basecalling on three samples and observed only 48, 50, or 65% of the reads passed the filters. When we performed super‐high accuracy basecalling on the same fast5 files (at the end of the sequencing runs), we obtained more than 80% of reads passing filters (Figure 1F). Next, we compared high‐accuracy basecalling (performed during the sequencing) and super‐high accuracy basecalling (performed separately from the sequencing). While the fast basecalling did not give a high percentage of good quality reads, high accuracy and super‐high accuracy basecalling gave similar results with a median percentage of pass reads of about 75% (Figure 1G). Even though we observed the same results with high accuracy and super‐high accuracy basecalling, we analyzed our samples with super‐high accuracy once the sequencing runs had been completed. We also observed the percentage of pass reads was not influenced by the throughput (Figure S2B). Similarly, neither the percentage of reads in the target (Figure S2C) nor the enrichment (Figure S2D) were influenced by the sequencing throughput. The enrichment is directly linked to the percentage of reads in the target (Figure S2E). Moreover, enrichment had an important influence on the coverage of target genes as we obtained higher coverages with higher enrichments (Figure S2F). Finally, by using adaptive sampling, we obtained a mean coverage of shallow whole genome sequencing from the rejected gene of 2.04× (0.76–4.29), whereas the mean coverage on the genes contained in the gene selection file (also called manifest) was 14.9× (4.71–35.08), corresponding to a mean enrichment of 7.26× (3.74–9.42; Figure 1H; Figure S2G).

### 3.2 ONT adaptive sampling improves the characterization of LSR

**FIGURE 2:**
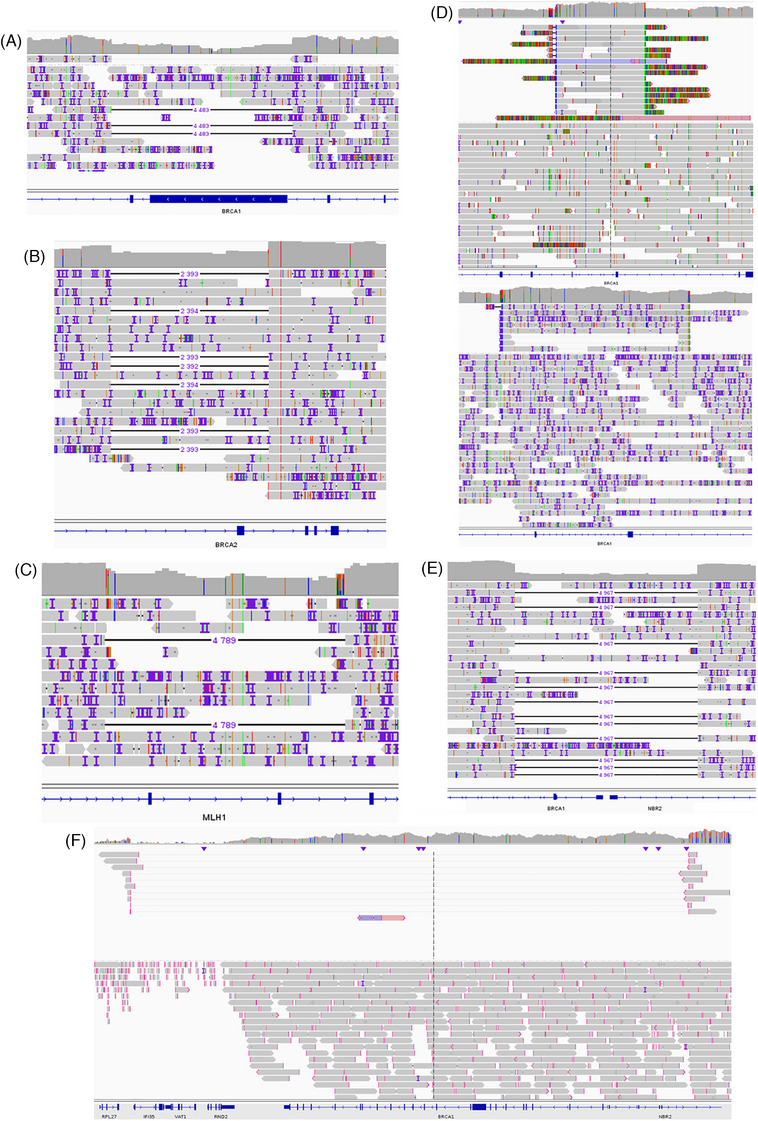
Integrative Genomics Viewer (IGV) visualization of LSR obtained with ONT sequencing. (A) Sample #5 had a 4483 bp deletion carrying exons 11 and 12 of the *BRCA1* gene. (B) Sample #22 had a 2393 bp deletion bringing exon 4 of the *BRCA2* gene. (C) Sample #17 harboured a 4789 deletion in the *MLH1* gene corresponding to a deletion of exons 4 and 5. (D) Samples #9 (up) and #10 (down) were relatives who both had a 6268 bp amplification in the *BRCA1* gene. This amplification corresponds to exon 13 duplication in relation to reference NM_7294, but is in fact a duplication in tandem of both exons 12 and 13 related to reference NM_7300. (E) Sample #7 had a deletion of 4967 bp carrying out the promoter, exons 1 and 2 of the *BRCA1* gene, but also the promoter and exon1 of the *NBR2* gene. (F) Sample #8 presented a large deletion of 139 603 bp bringing out five genes: *NBR2*, *BRCA1*, *RND2*, *VAT*, and *IFI35*. ONT, Oxford Nanopore Technologies.

Among our 30 samples, 11 carried an LSR on different genes detected by MLPA (Multiplex Ligation‐dependent Probe Amplification; Table 2). As ONT sequencing allows the analysis of long DNA fragments, we wondered whether the sequencing of complete genes from a bed file could detect the same LSR as MLPA. As expected, all LSR were also detected with ONT adaptive sampling (Table 2). Moreover, with the sequencing of introns, we were also able to identify the exact start and stop coordinates of each LSR (Table 2), and the exact size of deletions or amplifications. For example, sample #5 harbored a deletion of exons 11 and 12 of *BRCA1* by MLPA (Figure S3A). With adaptive sampling, we identified a deletion of 4483 bp from intron 10 to intron 12 (Figure 2A, Table 2). We also perfectly characterized two other deletions of exon 4 in *BRCA2* (Figure S3B) and exons 4 and 5 in *MLH1* (Figure S3C). Indeed, we observed a 2393 bp deletion, from intron 3 to intron 4, carrying exon 4 of *BRCA2* (Figure 2B, Table 2), and a deletion of 4789 bp deletion from intron 3 to intron 5, carrying exons 4 and 5 of *MLH1* (Figure 2C, Table 2). In addition to identifying some well‐known LSR, we also better characterized some others. Indeed, a sample originally identified as having an exon 13 duplication in *BRCA1* (NM_7294; Figure S3D up), was characterized as a carrier of duplication of both exons 12 and 13 of *BRCA1* (NM_7300; Figure 2D up, Table 2). We confirmed this observation on another sample carrying the same alteration (Figure 2D down, Figure S3D down). Another sample was identified with a partial deletion of promoter and a complete deletion of exon 1a and exon 2 of *BRCA1* (Figure S3E). ONT sequencing showed a large deletion of 4967 bp carrying whole exons 1 and 2 of *BRCA1* but also exon 1 of the non‐coding protein *NBR2* gene (Figure 2E). Finally, we considered another sample harbouring a complete deletion of *BRCA1* (Supplemental_Fig_S3F). We confirmed the complete deletion of the *BRCA1* but observed this deletion was part of a larger deletion of 139 603 bp carrying over five complete genes *NBR2*, *BRCA1*, *RND2*, *VAT*, and *IFI35* (Figure 2F).

**TABLE 2:** Comparison of large‐scale rearrangements observed by MLPA and nanopore sequencing.

Samples	Gene	Exons	Rearrangements observed with MLPA	Rearrangements observed with nanopore	Start	Stop	Size (bp)
#2	*BRCA1*	16, 17, 18, 19, 20, 21, 22, 23	c.(4675+1_4676‐1)_(5467+1_5468‐1)del	c.4676‐1720_5467+1434del	Chr17: 41 198 226	Chr17: 41 224 975	26 749
#4	*BRCA1*	5, 6, 7	c.(134+1_135‐1)_(441+1_442‐1)del	c.134‐530_441+1760del	Chr17: 41 254 380	Chr17: 41 259 079	4700
#5	*BRCA1*	11, 12	c.(670+1_671‐1)_(4185+1_4186‐1)del	c.671‐115_4185+447del	Chr17: 41 242 514	Chr17: 41 246 992	4479
#7	*BRCA1*	1, 2	c.(?_‐232)_(80+1_81‐1)del	c.1‐3845_80+1042del	Chr17: 41 274 992	Chr17: 41 279 958	4967
#8	*BRCA1*	All	c.(?_‐30)_(*220_?)	c.1‐21476_5592+39708del	Chr17: 41 157 987	Chr17: 41 297 589	139 603
#9	*BRCA1*	13	c.(4185+1_4186‐1)_(4357+1_4358‐1)dup	c.4186‐1879_4357+4217dup	Chr17: 41 230 204	Chr17: 41 236 471	6268
#10	*BRCA1*	13	c.(4185+1_4186‐1)_(4357+1_4358‐1)dup	c.4186‐1879_4357+4217dup	Chr17: 41 230 204	Chr17: 41 236 471	6268
#14	*BRCA1*	23	c.(5406+1_5407‐1)_(5467+1_5468‐1)del	c.5470‐754_5530+1437del	Chr17: 41 198 193	Chr17: 41 200 474	2282
#17	*MLH1*	4, 5	c.(306+1_307‐1)_(453+1_454‐1)del	c.307‐856_453+1270del	Chr3: 37 045 036	Chr3: 37 049 824	4789
#22	*BRCA2*	4	c.(316+1_317‐1)_(425+1_426‐1)del	c.317‐1920_425+364del	Chr13: 32 897 293	Chr13: 32 899 685	2393
#26	*BRCA2*	20	c.(8487+1_8488‐1)_(8632+1_8633‐1)del	c.8488‐170_8632+66del	Chr13: 32 944 923	Chr13: 32 945 303	381

### 3.3 ONT adaptive sampling accurately detects single nucleotide variants

**FIGURE 3:**
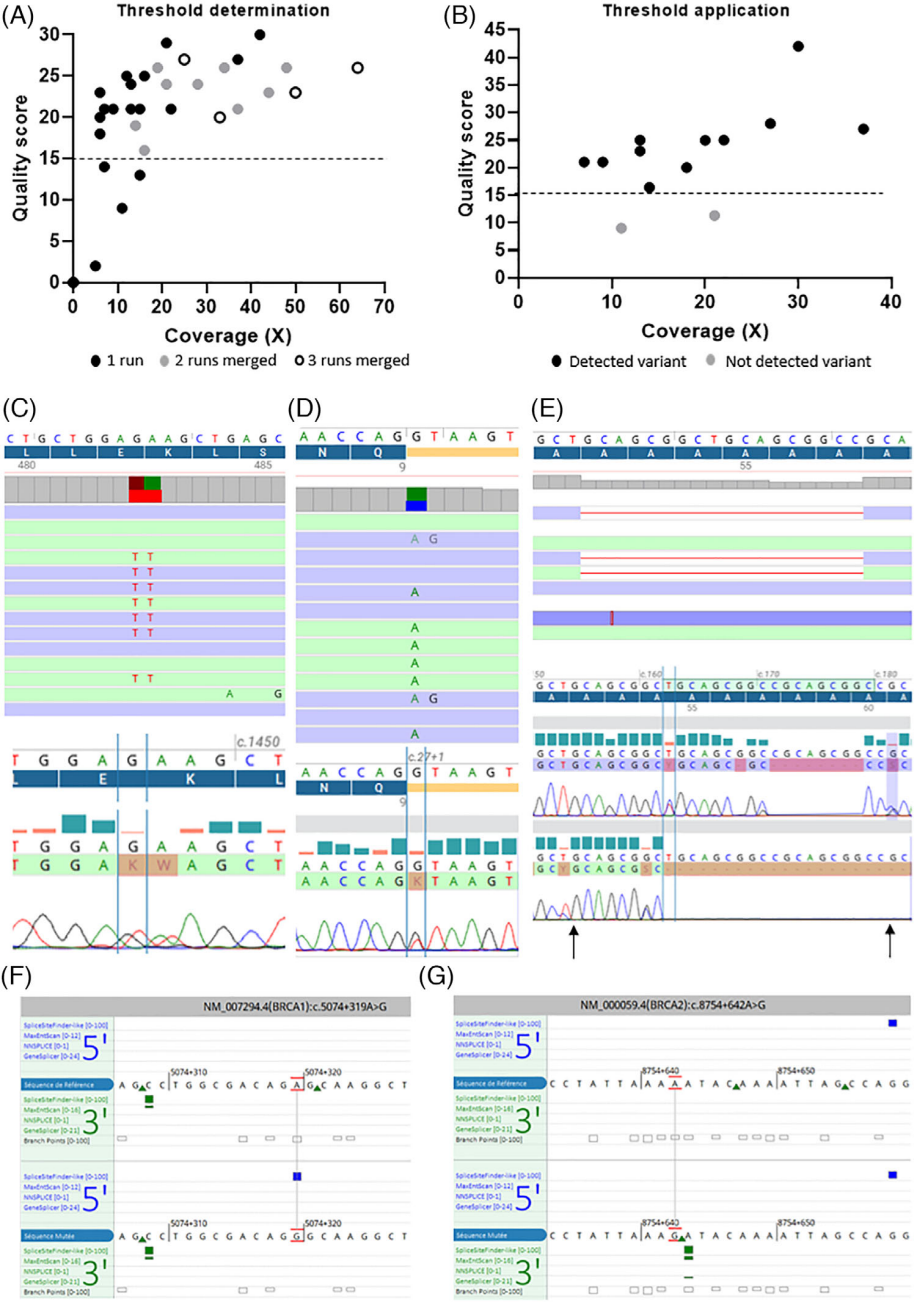
ONT adaptive sampling sequencing allows accurate detection of SNV. (A) To perform the determination of some thresholds, eight samples were sequenced two or three times. Obtained data were used on their own (black dots) but were also merged in silico to create runs with higher generated data from two (grey dots) and three (white dots) single runs. Thanks to these multiple runs, we observed that, for the observed SNV, when the coverage was higher than 10×, the quality score was improved. A quality threshold defined at 14, associated with coverage over 10×, seemed adapted to validate SNV with ONT sequencing. (B) By applying these thresholds, 11 well‐known variants detected with short‐read sequencing were validated (black dots), whereas only two (grey dots) were not. Both these variants had low quality despite a coverage higher than 10 due to frameshift induced by the mutations. (C–E) We confirmed by Sanger sequencing some SNV detected with ONT sequencing and selected with the quality and coverage criteria we defined. We confirmed the double mutation c.703_704delinsAA in the *GPT* gene (C), the point splicing mutation c.27+1G > T in the *CFAP126* gene (D), and the deletion c.162_179del in the *MSH3* gene (E). Arrows define the start and the stop of the deletion. (F, G) In sample #28, without pathological or likely pathological mutation in genes analyzed by short‐read sequencing despite a family history of cancer, the analysis of deep intron sequences allowed the detection of variations that could create a new 5′ splicing site in *BRCA1* gene (F) or a new 3′ splicing site in *BRCA2* gene (G). ONT, Oxford Nanopore Technologies; SNV, single nucleotide variations.

With the previous chemistry of ONT sequencing, the detection of SNV showed a high error rate. In order to test the accuracy of the new chemistry, we analyzed SNV detection on 15 samples with likely pathogenic/pathogenic (*n* = 15) or unknown (*n* = 2) variants previously detected by short‐read sequencing and 4 samples without any variants detected by short‐read sequencing (Table 3). All pathogenic variants except two were listed in the vcf files obtained after the alignment and variant calling. By focusing on the alignment files (bam format) for the two undetected variants, we observed them directly with an allelic frequency between 25 and 40% (Table 3). The two variants were not bioinformatically detected, probably due to their nucleotide environment and the sequence complexity created by the mutations. Indeed, homopolymer regions and highly repeated regions are difficult to sequence. Despite important advances in chemistry, sequencing could create artefacts, whatever the technology. For that, it is important to be able to discriminate true from false SNV. With short read sequencing, minimum coverage, and quality scores are now well established to validate variants.^22^ With ONT sequencing, especially with adaptive sampling and sequencing of native DNA, these quality criteria are not established yet. That is why we performed some analyses to find the minimum coverage and quality score allowing confidence in SNV detection. Ten samples were sequenced multiple times (2 or 3 times) due to low sequencing throughput. We performed variant analysis on the 21 runs and the 14 concatenated files obtained from the different runs. For each sample, we selected a specific SNV and followed its coverage and quality score for each run and each concatenation, representing a total of 35 SNV. We know the improvement of sequencing throughput increases coverage. Herein, we observed increase in coverage tended to improve the quality score of SNV for a majority of samples (Figure 3A). Moreover, we observed a main the part of the dots had a quality higher than 14. Then, we applied this quality threshold to the variants detected with SeqOne's bioinformatics pipeline and listed in Table 3. Eleven of 13 (85%) variants presented a quality higher than 14 (Figure 3B). The SNV of the 2 variants with a lower quality score were insertions. Nucleotide insertion or deletion‐induced sequencing or bioinformatics issues as both variations had quality scores below 14. These variants, not detected with our bioinformatics pipeline, were all frameshift mutations.

**TABLE 3:** Comparison of nucleotide variations observed by short read sequencing and Oxford Nanopore Technologies sequencing.

Samples	Gene	Mutations observed		Allele frequency/read depth with short read sequencing	Allele frequency/read depth with nanopore sequencing	Classification	Quality score
#1	*BRCA1*	c.5266dup	p.(Gln1756ProfsTer74)	43.4%/491	54.5%/11	Pathogenic	9
#3	*MLH1*	c.1852_1854delAAG	p.(Lys618del)	51.6%/184	40%^a^/10	Pathogenic	Not available
#6	*PALB2*	c.2835‐1G > C	*Splicing*	45.1%/91	21.4%/14	Pathogenic	16.41
#11	*BRCA2*	c.2376C > A	p.(Tyr792Ter)	48.7%/1088	50%/30	Pathogenic	42
#12	*BRCA1*	c.5044_5048delinsT	p.(Glu1682Ter)	44.4%/1912	80%/10	Pathogenic	15
#13	*BRCA2*	c.5645C > A	p.(Ser1882Ter)	48.7%/3149	53.8%/18	Pathogenic	20
#15	No alteration observed						
#16	*BRCA1*	c.1116G > A	p.(Trp372Ter)	46.8%/1589	60%/20	Pathogenic	24.94
#18	*BRCA2*	c.8586_8589delinsTTCACTAAAAG	p.(Glu2863SerfsTer8)	35.5%/1112	33.3%/21	Pathogenic	11.29
#19	*MLH1*	c.2059C > T	p.(Arg687Trp)	52.4%/338	28.6%/37	Pathogenic	27
#20	*BRCA1*	c.5154G > A	p.(Trp1718Ter)	42.9%/655	53.8%/13	Pathogenic	25
#21	*MLH1*	c.1178T > C	p.(Leu393Pro)	48.8%/863	44%/9	Pathogenic	21
#23	*MLH1*	c.2262del	p.(Arg755GlyfsTer28)	44.3%/1434	57.1%/7	Unknown	21
#24	*BRCA1* *PALB2*	c.1016dup c.529A > T	p.(Val340GlyfsTer6) p.(Lys177Ter)	46.6%/223 54.1%/109	25%^a^/24 51.9%/27	Pathogenic Pathogenic	Not available 28
#25	*PALB2*	c.1438A > T	p.(Lys480Ter)	49.6%/964	36.4%/22	Pathogenic	25
#27	*BRCA2*	c.5065_5066del	p.(Ala1689LysfsTer5)	35.8%/542	42.4%/13	Unknown	23
#28	No alteration observed						
#29	No alteration observed						
#30	No alteration observed						

^a^ Single nucleotide variations only present in raw data.

Quality score threshold is not sufficient to validate a mutation and guidelines need to apply a minimum read depth. With short read sequencing, the minimum read depth is 30×. With ONT adaptive sequencing, native gDNA is sequenced without any PCR amplification. This specificity completely abolishes the appearance of PCR artefacts. Based on this specificity, we thought a read depth lower than 30× could be applied. We observed a majority of good‐quality mutations had a coverage higher than 10× (Figure 3A). By applying these filters (quality > 14 and coverage > 10×), we were able to detect 82.9% (Figure 3B) of already known variants present in Table 3.

Then, we tested if we could confirm, with Sanger sequencing, variants selected by applying both thresholds on genes that were not analyzed by short‐read sequencing. We randomly selected six variants in six different genes (Table S2) representing different types of alterations (small deletion, transition, transversion, double point mutations) for which we designed specific PCR primers for Sanger sequencing. All variants were confirmed by the gold standard: the double mutation c.703_704delinsAA in the *GPT* gene (Figure 3C), the splicing mutation c.27+1G > T in the *CFAP126* gene (Figure 3D), the deletion c.162_179del in the *MSH3* gene (Figure 3E), and three other point mutations in *ANKRD26*, *TFKC*, and *BLM* genes (Figure S4).

Finally, the use of adaptive sampling allows the analysis of whole gene sequences and therefore introns. In a sample with a family cancer history but without any alteration in coding sequences of cancer‐predisposing genes analyzed by short‐read sequencing, we observed some deep intron variants meeting our quality and coverage criteria. After checking in silico the splicing impact of these variants, we observed that two of them could create new alternative splicing sites in either *BRCA1* with a new 5′ splicing site (Figure 3F) or *BRCA2* with a new 3′ splicing site (Figure 3G). These variations need to be characterized with specific methods as minigene experiments. Unfortunately, we were not able to study their impact as it was impossible to obtain new blood samples dedicated to RNA analysis from these patients.

## 4 DISCUSSION

In this work, we validated the technical performances of adaptive sampling from germline DNA samples. We set up the amount of gDNA to reach the best yield of library preparation. We showed age of the DNA did not impact library preparation yield, and sequencing throughput was directly impacted by the number of available pores before starting sequencing. Finally, the use of high accuracy or super‐high accuracy basecalling was mandatory to obtain a higher enrichment of targets. In this way, we showed that the limitations and drawbacks discussed in the work from Loose et al.^2^ have been overcome with the R10.4.1 MinION flowcells with the Q20+ chemistry on a GridION device. Indeed, within the originator publication, the authors pointed out that sequencing and alignment speeds needed to be improved. With the new version of the MINknow control software and the GridION calculation capacities associated with the new R10 flow cells, the supplier has announced the decision time is below 1 s corresponding to 400 sequenced nucleotides.

Long‐read sequencing is well known for giving a high resolution for the detection of structural variations but, only one case report showed the usefulness of adaptive sampling on *BRCA1* gene.^23^ Among the 30 samples we studied, we were able to perfectly determine the start and stop coordinates of each LSR. This exact determination allowed us to show that a duplication of exon 13 (reference NM_7294) in *BRCA1*, detected with MLPA, was a duplication in tandem of both exons 12 and 13 (reference NM_7300). For another sample that carried a complete *BRCA1* gene deletion, we highlighted this deletion was part of a larger deletion of 139,603 bp carrying over 4 other genes: *NBR2*, *RND2*, *VAT*, and *IFI35*. The knowledge of this specific large deletion gave a new look at cancer risk. Indeed, the *NBR2* gene encodes a long noncoding RNA that suppresses tumour development through regulation of adenosine monophosphate‐activated protein kinase activation.^24^ Another sample had a large deletion of the promoter and both exons 1 and 2 of *BRCA1* gene, also carrying the promoter and the first exon of *NBR2* gene. Consequently, the loss of both *BRCA1* and *NBR2* genes could increase the risk of cancer development and impact the clinical follow‐up. Based on our observations, long read adaptive sampling has a better resolution than MLPA which is largely used in molecular diagnosis labs because of its low cost. Adaptive sampling has the same resolution as whole genome sequencing, which is the gold standard. Consequently, adaptive sampling could be a better tool than MLPA for the detection and characterization of LSR in routine molecular diagnosis labs, as it has the same resolution as whole genome sequencing, but at a lower cost.

The most frequent alteration in cancer‐predisposing genes is SNV. A case report describing the comparison of short‐read and long‐read adaptive sampling showed a perfect concordance for a pathogenic duplication in the *RB1* gene between both technologies.^25^ Herein, we tested 19 samples previously analyzed with a 35‐gene panel by short‐read sequencing, representing 16 well‐known alterations. We confirmed, on raw data (bam files), that all SNV were present after ONT sequencing, but two of these were not listed after applying automatic variant calling. This was due to the high complexity of these SNV that were either present in highly repeated regions or induced mononucleotidic repeats, making the sequence difficult to analyze. Consequently, both basecalling and bioinformatics pipelines need to be improved.

We also tested whether SNV detected on genes not previously analyzed could be confirmed by Sanger sequencing. By applying filters based on a quality score higher than 14, and a coverage higher than 10×, we confirmed all selected SNV (three different point mutations, two consecutive point mutations, and a small deletion) with Sanger sequencing. This is the first time that SNV detected by long‐read adaptive sampling was confirmed by Sanger sequencing. This suggests that, with specific coverage (>10×) and quality (>14) filters, we can be confident in SNV detected from native DNA by ONT sequencing. Adaptive sampling selects genomic regions throughout the sequencing process, and, contrary to capture‐based enrichment used with short‐read sequencing, the whole sequence of genes is sequenced. This highlighted a high number of unknown significance variants as deep intron variants have not been studied routinely yet. Indeed, with short‐read sequencing, the only way to catch these deep intron variations is whole genome sequencing^26^, ^27^ and/or whole transcriptome sequencing^28^, ^29^ that are not currently used in routine molecular diagnosis laboratories. Although these variants are deeply intronic and not currently sought, they may be pathogenic.^30^, ^31^ Adaptive sampling could be a good alternative between short‐read panel and whole genome or transcriptome sequencing. As an example, one of our samples without known alteration but with a family cancer history, had two deep intron variants predicted, in silico, as creating new alternative splicing sites in *BRCA1* (new 5′ splicing site), and *BRCA2* (new 3′ splicing site). Further analyses should be performed to determine whether these new splicing sites could impact the splicing of BRCA1 or BRCA2 mRNA, for example with minigene experiments, but the systematic analysis of non‐coding sequences of cancer‐predisposing genes could improve the efficiency of molecular diagnosis.

Herein, we only focused on LSR and SNV, but ONT sequencing can also detect methylation of cytosine from native DNA.^32^ Even if methylation status is not usually studied routinely, some methylation marks can be inherited and play a role in cancer predisposition.^33^, ^34^ These marks are not necessarily located in promoters of predisposing genes and therefore could be missed. However, adaptive sampling selects regions of interest throughout sequencing. From rejected reads giving a low pass whole genome sequencing, methylation or other parameters can be studied as genome‐wide SNP, or more specifically SNP involved in a polygenic risk score.^35^, ^36^, ^37^, ^38^

**FIGURE 4:**
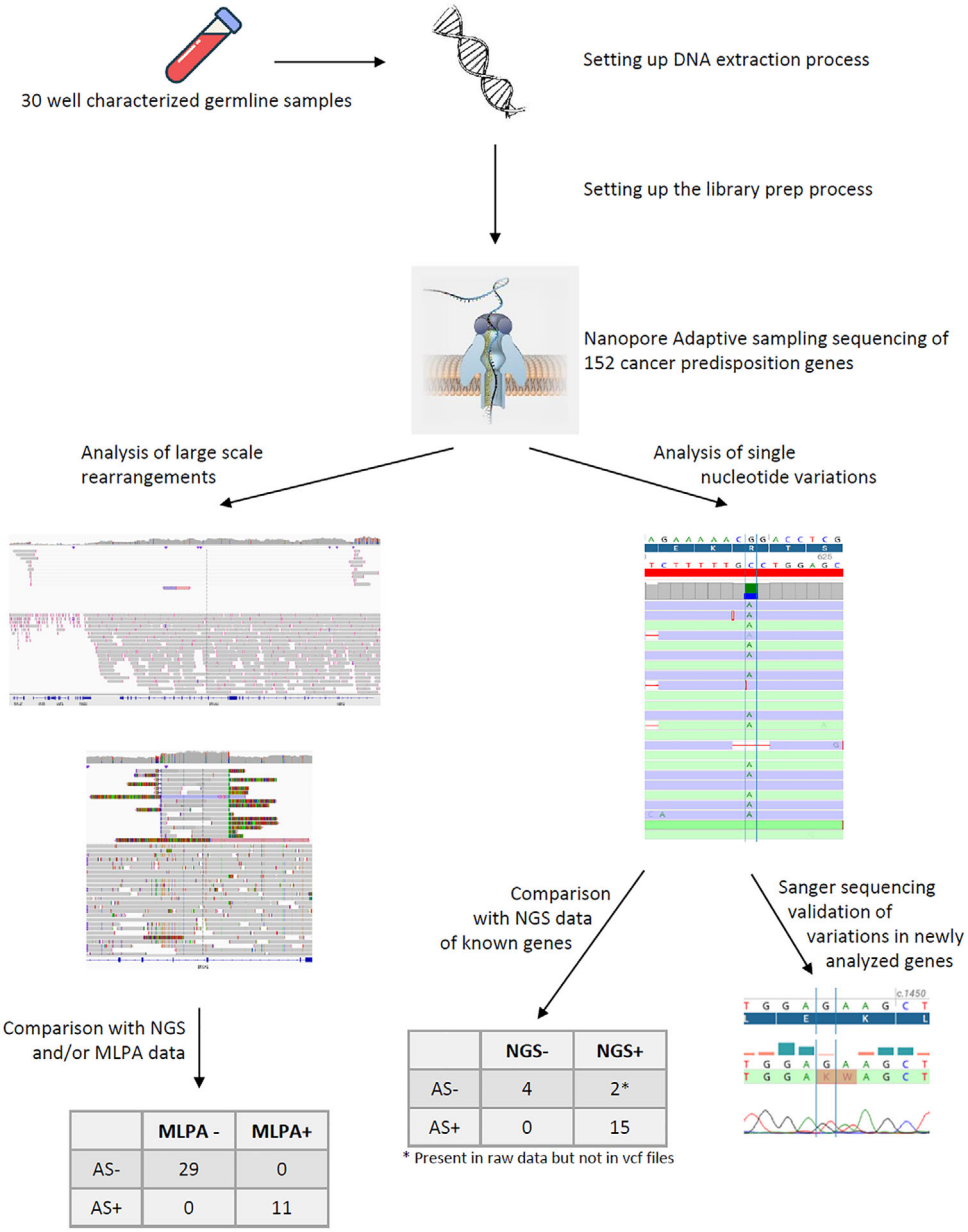
1, Nanopore adaptive sampling sequencing is reliable for the analysis of germline alterations by improving the characterization of LSR and detecting SNV even at low coverage. 2, The protocol we set up can be immediately used in routine molecular diagnosis lab for characterization of large‐scale rearrangements. LSR, large‐scale rearrangements; SNV, single nucleotide variations.

In conclusion, we showed that the ONT adaptive sampling sequencing workflow was easy to manage (Figure S5) and is suitable for the analysis of germline alterations. It improves the characterization of LSR and is able to detect SNV even at low coverage (>10×; Figure 4). The multiplexing of samples on PromethION flow cells will increase the coverage, improve the quality of variant detection, and reduce the cost of the analysis. Moreover, the flexibility of ONT sequencing (from a single sample to numerous samples at the same time) would improve the turn‐around time. Nevertheless, an improvement in the detection of variants located in homopolymer regions should be considered. Once such an improvement is done, larger comparison studies between adaptive sampling and short read sequencing should be performed to obtain performance data (sensitivity, specificity, positive predicting value, negative predicting value…), and, then to adapt international guidelines to third‐generation sequencing for germline diagnosis.

## AUTHOR CONTRIBUTIONS

Study design and supervision: Romain Boidot. Data curation: Romain Boidot. Investigation: Sandy Chevrier. Result analysis: Romain Boidot, Corentin Richard, Marie Mille, and Denis Bertrand. Result visualization: Romain Boidot. Manuscript writing: Romain Boidot.

## CONFLICT OF INTEREST STATEMENT

The cost of reagents was supported by ONT. MM and DB are employees of SeqOne Genomics. RB is a paid consultant of SeqOne Genomics.

## ETHICS STATEMENT AND CONSENT TO PARTICIPATE

All patients signed an informed consent to participate in the trial. The trial protocol was approved by an institutional review committee and by the relevant French regional ethical committee (*Comité de protection des personnes Est*).

## CONSENT FOR PUBLICATION

All patients, by giving their consent to participate, have given their consent for publication.

## Supporting information



Supporting Information

Supporting Information

Supporting Information

Supporting Information

Supporting Information

Supporting Information

Supporting Information

Supporting Information

## Data Availability

Genomic data may be shared upon reasonable request to the corresponding author in accordance with the French law on genomic data.

## References

[1] van Dijk EL , Jaszczyszyn Y , Naquin D , Thermes C , The third revolution in sequencing technology. Trends Genet. 2018;34(9):666‐681. doi:10.1016/j.tig.2018.05.008 29941292

[2] Loose M , Malla S , Stout M , Real‐time selective sequencing using nanopore technology. Nat Methods. 2016;13(9):751‐754. doi:10.1038/nmeth.3930 27454285 PMC5008457

[3] Mighton C , Lerner‐Ellis JP , Principles of molecular testing for hereditary cancer. Genes Chromosomes Cancer. 2022;61(6):356‐381. doi:10.1002/gcc.23048 35436018

[4] Logsdon GA , Vollger MR , Eichler EE , Long‐read human genome sequencing and its applications. Nat Rev Genet. 2020;21(10):597‐614. doi:10.1038/s41576-020-0236-x 32504078 PMC7877196

[5] Begum G , Albanna A , Bankapur A , et al. Long‐read sequencing improves the detection of structural variations impacting complex non‐coding elements of the genome. Int J Mol Sci. 2021;22(4)doi:10.3390/ijms22042060 PMC7923155 33669700

[6] Conlin LK , Aref‐Eshghi E , McEldrew DA , Luo M , Rajagopalan R , Long‐read sequencing for molecular diagnostics in constitutional genetic disorders. Hum Mutat. 2022;43(11):1531‐1544. doi:10.1002/humu.24465 36086952 PMC9561063

[7] Li H , Minimap2: pairwise alignment for nucleotide sequences. Bioinformatics. 2018;34(18):3094‐3100. doi:10.1093/bioinformatics/bty191 29750242 PMC6137996

[8] Zheng Z , Li S , Su J , Leung AW , Lam TW , Luo R , Symphonizing pileup and full‐alignment for deep learning‐based long‐read variant calling. Nat Comput Sci. 2022;2(12):797‐803. doi:10.1038/s43588-022-00387-x 38177392

[9] Tan A , Abecasis GR , Kang HM , Unified representation of genetic variants. Bioinformatics. 2015;31(13):2202‐2204. doi:10.1093/bioinformatics/btv112 25701572 PMC4481842

[10] Hunt SE , Moore B , Amode RM , et al. Annotating and prioritizing genomic variants using the Ensembl variant effect predictor‐a tutorial. Hum Mutat. 2022;43(8):986‐997. doi:10.1002/humu.24298 34816521 PMC7613081

[11] Landrum MJ , Lee JM , Benson M , et al. ClinVar: improving access to variant interpretations and supporting evidence. Nucleic Acids Res. 2018;46(D1):D1062‐D1067. doi:10.1093/nar/gkx1153 29165669 PMC5753237

[12] Chen S , Francioli LC , Goodrich JK , et al. A genomic mutational constraint map using variation in 76,156 human genomes. Nature. 2024;625(7993):92‐100. doi:10.1038/s41586-023-06045-0 38057664 PMC11629659

[13] Amberger JS , Bocchini CA , Scott AF , Hamosh A , OMIM.org: leveraging knowledge across phenotype‐gene relationships. Nucleic Acids Res. 2019;47(D1):D1038‐D1043. doi:10.1093/nar/gky1151 30445645 PMC6323937

[14] Richards S , Aziz N , Bale S , et al. Standards and guidelines for the interpretation of sequence variants: a joint consensus recommendation of the American College of Medical Genetics and Genomics and the Association for Molecular Pathology. Genet Med. 2015;17(5):405‐424. doi:10.1038/gim.2015.30 25741868 PMC4544753

[15] Smolka M , Paulin LF , Grochowski CM , et al. Detection of mosaic and population‐level structural variants with Sniffles2. Nat Biotechnol. 2024;42:1571–1580. doi:10.1038/s41587-023-02024-y 38168980 PMC11217151

[16] MacDonald JR , Ziman R , Yuen RK , Feuk L , Scherer SW , The Database of Genomic Variants: a curated collection of structural variation in the human genome. Nucleic Acids Res. 2014;42(Database issue):D986‐992. doi:10.1093/nar/gkt958 24174537 PMC3965079

[17] MedGen LoudenDN , : NCBI's portal to information on medical conditions with a genetic component. Med Ref Serv Q. 2020;39(2):183‐191. doi:10.1080/02763869.2020.1726152 32329672

[18] Rehm HL , Berg JS , Brooks LD , et al. ClinGen–the Clinical Genome Resource. N Engl J Med. 2015;372(23):2235‐2242. doi:10.1056/NEJMsr1406261 26014595 PMC4474187

[19] Bragin E , Chatzimichali EA , Wright CF , et al. DECIPHER: database for the interpretation of phenotype‐linked plausibly pathogenic sequence and copy‐number variation. Nucleic Acids Res. 2014;42(Database issue):D993‐D1000. doi:10.1093/nar/gkt937 24150940 PMC3965078

[20] Collins RL , Brand H , Karczewski KJ , et al. A structural variation reference for medical and population genetics. Nature. 2020;581(7809):444‐451. doi:10.1038/s41586-020-2287-8 32461652 PMC7334194

[21] Riggs ER , Andersen EF , Cherry AM , et al. Technical standards for the interpretation and reporting of constitutional copy‐number variants: a joint consensus recommendation of the American College of Medical Genetics and Genomics (ACMG) and the Clinical Genome Resource (ClinGen). Genet Med. 2020;22(2):245‐257. doi:10.1038/s41436-019-0686-8 31690835 PMC7313390

[22] Crooks KR , Farwell Hagman KD , Mandelker D , et al. Recommendations for next‐generation sequencing germline variant confirmation: a joint report of the Association for Molecular Pathology and National Society of Genetic Counselors. J Mol Diagn. 2023;25(7):411‐427. doi:10.1016/j.jmoldx.2023.03.012 37207865

[23] Filser M , Schwartz M , Merchadou K , et al. Adaptive nanopore sequencing to determine pathogenicity of BRCA1 exonic duplication. J Med Genet. 2023;60(12):1206‐1209. doi:10.1136/jmg-2023-109155 37263769 PMC10715497

[24] Xiao ZD , Liu X , Zhuang L , Gan B , NBR2: a former junk gene emerges as a key player in tumor suppression. Mol Cell Oncol. 2016;3(4):e1187322. doi:10.1080/23723556.2016.1187322 27652330 PMC4972102

[25] Nakamichi K , Stacey A , Mustafi D , Targeted long‐read sequencing allows for rapid identification of pathogenic disease‐causing variants in retinoblastoma. Ophthalmic Genet. 2022;43(6):762‐770. doi:10.1080/13816810.2022.2141797 36325802

[26] Walker S , Lamoureux S , Khan T , et al. Genome sequencing for detection of pathogenic deep intronic variation: a clinical case report illustrating opportunities and challenges. Am J Med Genet A. 2021;185(10):3129‐3135. doi:10.1002/ajmg.a.62389 34159711

[27] Dirix M , Gribouval O , Arrondel C , et al. Overcoming the challenges associated with identification of deep intronic variants by whole genome sequencing. Clin Genet. 2023;103(6):693‐698. doi:10.1111/cge.14305 36705481

[28] Cummings BB , Marshall JL , Tukiainen T , et al. Improving genetic diagnosis in Mendelian disease with transcriptome sequencing. Sci Transl Med. 2017;9(386):eaal5209. doi:10.1126/scitranslmed.aal5209 28424332 PMC5548421

[29] Chorin O , Yachelevich N , Mohamed K , et al. Transcriptome sequencing identifies a noncoding, deep intronic variant in CLCN7 causing autosomal recessive osteopetrosis. Mol Genet Genomic Med. 2020;8(10):e1405. doi:10.1002/mgg3.1405 32691986 PMC7549584

[30] Vaz‐Drago R , Custodio N , Carmo‐Fonseca M , Deep intronic mutations and human disease. Hum Genet. 2017;136(9):1093‐1111. doi:10.1007/s00439-017-1809-4 28497172

[31] Senju C , Nakazawa Y , Oso T , et al. Deep intronic founder mutations identified in the ERCC4/XPF gene are potential therapeutic targets for a high‐frequency form of xeroderma pigmentosum. Proc Natl Acad Sci USA. 2023;120(27):e2217423120. doi:10.1073/pnas.2217423120 37364129 PMC10318981

[32] Simpson JT , Workman RE , Zuzarte PC , David M , Dursi LJ , Timp W , Detecting DNA cytosine methylation using nanopore sequencing. Nat Methods. 2017;14(4):407‐410. doi:10.1038/nmeth.4184 28218898

[33] Fernandez JA , Patnaik MM , Germline abnormalities in DNA methylation and histone modification and associated cancer risk. Curr Hematol Malig Rep. 2022;17(4):82‐93. doi:10.1007/s11899-022-00665-5 35653077

[34] Joo JE , Dowty JG , Milne RL , et al. Heritable DNA methylation marks associated with susceptibility to breast cancer. Nat Commun. 2018;9(1):867. doi:10.1038/s41467-018-03058-6 29491469 PMC5830448

[35] Torkamani A , Wineinger NE , Topol EJ , The personal and clinical utility of polygenic risk scores. Nat Rev Genet. 2018;19(9):581‐590. doi:10.1038/s41576-018-0018-x 29789686

[36] Lewis CM , Vassos E , Polygenic risk scores: from research tools to clinical instruments. Genome Med. 2020;12(1):44. doi:10.1186/s13073-020-00742-5 32423490 PMC7236300

[37] Hao L , Kraft P , Berriz GF , et al. Development of a clinical polygenic risk score assay and reporting workflow. Nat Med. 2022;28(5):1006‐1013. doi:10.1038/s41591-022-01767-6 35437332 PMC9117136

[38] Nakamura W , Hirata M , Oda S , et al. Assessing the efficacy of target adaptive sampling long‐read sequencing through hereditary cancer patient genomes. NPJ Genom Med. 2024;9(1):11. doi:10.1038/s41525-024-00394-z 38368425 PMC10874402

