# Supplementary material for: Nanopore adaptive sampling accurately detects nucleotide variants and improves the characterization of large‐scale rearrangement for the diagnosis of cancer predisposition
Source: Clin Transl Med. 2025 Jan 9;15(1):e70138. doi: 10.1002/ctm2.70138 (PMC11714230; doi:10.1002/ctm2.70138)
Supplement: Supplementary file 6 — Supporting Information [file CTM2-15-e70138-s005.docx]

Supplementary Table S1: Detail of genes present in the adaptive sampling bed file

| Gene | Related disease | Level of risk | chromosome | Start | stop |
| --- | --- | --- | --- | --- | --- |
| *ABRAXAS1* | Breast | Low / research | chr4 | 83,449,517 | 83,495,100 |
| *ANKRD26* | Leukaemia | Medium / Low w high frequency | chr10 | 26,994,116 | 27,110,494 |
| *ACD* | Leukaemia | Low / research | chr16 | 67,647,511 | 67,670,260 |
| *APC* | Digestive tract  CNS, Paediatric hereditary, Pancreas | High  Low / research | chr5 | 112,727,884 | 112,856,239 |
| *ATG2B* | Leukaemia | Low / research | chr14 | 96,269,194 | 96,373,341 |
| *ATM* | Breast, Digestive tract, Pancreas, Prostate  Ovary, Paediatric hereditary | Medium / Low w high frequency  Low / research | chr11 | 108,213,066 | 108,379,102 |
| *ATR* | Breast, Leukaemia, Ovary | Low / research | chr3 | 142,439,234 | 142,588,733 |
| *AXIN2* | Digestive tract | Medium / Low w high frequency | chr17 | 65,518,562 | 65,571,648 |
| *BAP1* | Kidney  Skin | Medium / Low w high frequency  High | chr3 | 52,391,007 | 52,420,008 |
| *BARD1* | Breast, Ovary, Prostate | Low / research | chr2 | 214,715,645 | 214,819,683 |
| *BLM* | Digestive tract, Leukaemia  Paediatric hereditary | Medium / Low w high frequency  Low / research | chr15 | 90,707,345 | 90,826,166 |
| *BMPR1A* | Digestive tract | High | chr10 | 86,746,618 | 86,937,969 |
| *BRCA1* | Breast, Ovary, Prostate  Pancreas  Skin | High  Medium / Low w high frequency  Low / research | chr17 | 43,034,294 | 43,135,364 |
| *BRCA2* | Breast, Ovary, Prostate  Pancreas  CNS, Skin | High  Medium / Low w high frequency  Low / research | chr13 | 32,305,085 | 32,410,268 |
| *BRIP1* | Breast, CNS, Ovary, Pancreas, Prostate | Low / research | chr17 | 61,669,138 | 61,873,528 |
| *CDH1* | Breast  Digestive tract  Ovary | High  Medium / Low w high frequency  Low / research | chr16 | 68,727,291 | 68,845,537 |
| *CDK4* | Skin | High | chr12 | 57,737,726 | 57,762,310 |
| *CDKN1B* | CNS | Low / research | chr12 | 12,675,497 | 12,732,325 |
| *CDKN1C* | Kidney | Medium / Low w high frequency | chr11 | 2,873,212 | 2,895,771 |
| *CDKN2A* | Pancreas  Skin | Medium / Low w high frequency  High | chr9 | 21,957,751 | 22,004,392 |
| *CDKN2B* | Skin | High | chr9 | 21,992,902 | 22,019,305 |
| *CEBPA* | Leukaemia | Medium / Low w high frequency | chr19 | 33,289,933 | 33,312,534 |
| *CFTR* | Pancreas | Medium / Low w high frequency | chr7 | 117,470,025 | 117,678,665 |
| *CHEK2* | Breast, Prostate  Digestive tract, Endocrin, Ovary, Pancreas, Prostate | Medium / Low w high frequency  Low / research | chr22 | 28,677,742 | 28,751,820 |
| *CREBBP* | Leukaemia, Paediatric hereditary | Low / research | chr16 | 3,715,053 | 3,890,713 |
| *CTC1* | Leukaemia | Low / research | chr17 | 8,214,814 | 8,258,056 |
| *CTNNA1* | Digestive tract | Low / research | chr5 | 138,743,425 | 138,945,034 |
| *DDX41* | Leukaemia | Medium / Low w high frequency | chr5 | 177,501,576 | 177,526,961 |
| *DICER1* | Endocrin, Paediatric hereditary | Low / research | chr14 | 95,076,243 | 95,168,010 |
| *DKC1* | Leukaemia | Low / research | chrX | 154,752,863 | 154,787,689 |
| *DNAH9* | Leukaemia | Low / research | chr17 | 11,588,469 | 11,979,748 |
| *DNAJC21* | Leukaemia | Low / research | chr5 | 34,919,558 | 34,968,964 |
| *ELANE* | Leukaemia | Low / research | chr19 | 841,013 | 866,247 |
| *EPCAM* | Breast  Digestive tract, Prostate  Ovary, Pancreas | Low / research  High  Medium / Low w high frequency | chr2 | 47,359,310 | 47,397,020 |
| *ERCC6L2* | Leukaemia | Low / research | chr9 | 95,865,690 | 96,028,447 |
| *ETV6* | Leukaemia | Medium / Low w high frequency | chr12 | 11,639,673 | 11,905,377 |
| *EVI1/MECOM* | Leukaemia | Low / research | chr3 | 169,073,507 | 169,673,712 |
| *EXT1* | Paediatric hereditary | Low / research | chr8 | 117,784,489 | 118,121,826 |
| *EXT2* | Paediatric hereditary | Low / research | chr11 | 44,085,677 | 44,261,962 |
| *FAN1* | Digestive tract | Low / research | chr15 | 30,893,864 | 30,953,085 |
| *FANCA* | CNS, Leukaemia, Paediatric hereditary | Low / research | chr16 | 89,727,548 | 89,826,647 |
| *FANCB* | CNS, Leukaemia, Paediatric hereditary | Low / research | chrX | 14,830,183 | 14,883,064 |
| *FANCC* | CNS, Leukaemia, Paediatric hereditary, Pancreas | Low / research | chr9 | 95,089,053 | 95,327,709 |
| *FANCD2* | CNS, Leukaemia, Paediatric hereditary | Low / research | chr3 | 10,016,436 | 10,111,932 |
| *FANCE* | CNS, Leukaemia | Low / research | chr6 | 35,442,337 | 35,477,102 |
| *FANCF* | CNS, Leukaemia, Paediatric hereditary | Low / research | chr11 | 22,612,532 | 22,635,823 |
| *FANCG* | CNS, Leukaemia, Paediatric hereditary, Pancreas | Low / research | chr9 | 35,063,855 | 35,089,969 |
| *FANCI* | CNS, Leukaemia | Low / research | chr15 | 89,233,978 | 89,327,131 |
| *FANCL* | CNS | Low / research | chr2 | 58,149,242 | 58,251,345 |
| *FANCM* | CNS | Low / research | chr14 | 45,125,929 | 45,210,890 |
| *FH* | Kidney  Endocrin | High  Low / research | chr1 | 241,487,602 | 241,529,755 |
| *FLCN* | Digestive tract  Kidney | Low / research  High | chr17 | 17,202,211 | 17,247,168 |
| *GALNT12* | Digestive tract | Low / research | chr9 | 98,797,670 | 98,860,081 |
| *GATA2* | Leukaemia | Medium / Low w high frequency | chr3 | 128,469,426 | 128,503,201 |
| *GPC2* | Kidney | Medium / Low w high frequency | chr7 | 100,159,605 | 100,187,381 |
| *GREM1* | Digestive tract | Medium / Low w high frequency | chr15 | 32,708,003 | 32,755,106 |
| *GSKIP* | Leukaemia | Low / research | chr14 | 96,353,451 | 96,397,286 |
| *HOXB13* | Breast  Prostate | Low / research  High | chr17 | 48,714,762 | 48,738,750 |
| *HRAS* | Paediatric hereditary | Low / research | chr11 | 522,241 | 545,576 |
| *IGF2* | Endocrin | Low / research | chr11 | 2,119,111 | 2,148,974 |
| *KRAS* | Leukaemia, Paediatric hereditary | Low / research | chr12 | 25,195,245 | 25,260,929 |
| *LIG4* | Leukaemia | Low / research | chr13 | 108,197,438 | 108,228,368 |
| *MAX* | Endocrin | Low / research | chr14 | 65,065,126 | 65,112,517 |
| *MBD4* | Leukaemia | Low / research | chr3 | 129,420,949 | 129,450,009 |
| *MC1R* | Skin | Medium / Low w high frequency | chr16 | 89,904,846 | 89,930,951 |
| *MEN1* | CNS, Endocrin, Paediatric hereditary | Low / research | chr11 | 64,793,515 | 64,820,686 |
| *MET* | Kidney | High | chr7 | 116,662,195 | 116,808,377 |
| *MITF* | Skin | Medium / Low w high frequency | chr3 | 69,729,464 | 69,978,332 |
| *MLH1* | Breast, CNS  Digestive tract, Prostate  Ovary, Pancreas | Low / research  High  Medium / Low w high frequency | chr3 | 36,983,517 | 37,060,846 |
| *MLH3* | Digestive tract  Prostate | Medium / Low w high frequency  High | chr14 | 75,003,774 | 75,061,467 |
| *MRE11* | Ovary, Pancreas, Prostate | Low / research | chr11 | 94,405,569 | 94,503,844 |
| *MSH2* | Breast, CNS  Digestive tract, Prostate  Ovary, Pancreas | Low / research  High  Medium / Low w high frequency | chr2 | 47,393,118 | 47,673,146 |
| *MSH3* | Digestive tract | Medium / Low w high frequency | chr5 | 80,644,651 | 80,886,815 |
| *MSH6* | Breast, CNS  Digestive tract, Prostate  Ovary, Pancreas | Low / research  High  Medium / Low w high frequency | chr2 | 47,773,144 | 47,816,953 |
| *MUTYH* | Digestive tract | High | chr1 | 45,319,241 | 45,350,115 |
| *NAF1* | Leukaemia | Low / research | chr4 | 163,118,668 | 163,176,890 |
| *NAPRT* | Leukaemia | Low / research | chr8 | 143,564,784 | 143,588,330 |
| *NBN* | Breast, Leukaemia, Ovary, Pancreas, Prostate | Low / research | chr8 | 89,914,514 | 89,994,682 |
| *NBR1* | Kidney | Low / research | chr17 | 43,160,481 | 43,221,689 |
| *NBR2* | Breast, Kidney | Low / research | chr17 | 43,115,551 | 43,163,649 |
| *NF1* | Breast, CNS, Endocrin, Paediatric hereditary | Low / research | chr17 | 31,084,976 | 31,387,675 |
| *NF2* | CNS, Paediatric hereditary | Low / research | chr22 | 29,593,632 | 29,708,598 |
| *NOP10* | Leukaemia | Low / research | chr15 | 34,331,719 | 34,353,136 |
| *NHP2* | Leukaemia | Low / research | chr5 | 178,139,463 | 178,163,885 |
| *NRAS* | Leukaemia, Paediatric hereditary | Low / research | chr1 | 114,694,468 | 114,726,771 |
| *NSD1* | CNS, Endocrin, Paediatric hereditary | Low / research | chr5 | 177,123,772 | 177,310,213 |
| *NTHL1* | Digestive tract | Medium / Low w high frequency | chr16 | 2,029,814 | 2,057,866 |
| *PALB2* | Breast  CNS, Prostate  Ovary, Pancreas | High  Low / research  Medium / Low w high frequency | chr16 | 23,593,164 | 23,651,310 |
| *PARN* | Leukaemia | Low / research | chr16 | 14,425,700 | 14,640,260 |
| *PAX5* | Leukaemia | Low / research | chr9 | 36,823,268 | 37,044,268 |
| *PHOX2B* | CNS, Endocrin, Paediatric hereditary | Low / research | chr4 | 41,734,081 | 41,758,725 |
| *PMS2* | Breast, CNS  Digestive tract, Prostate  Ovary, Pancreas | Low / research  High  Medium / Low w high frequency | chr7 | 5,960,924 | 6,019,049 |
| *POLD1* | Digestive tract | Medium / Low w high frequency | chr19 | 50,374,322 | 50,428,018 |
| *POLE* | Digestive tract | Medium / Low w high frequency | chr12 | 132,613,761 | 132,697,342 |
| *POLH* | Paediatric hereditary | Low / research | chr6 | 43,566,184 | 43,630,523 |
| *POT1* | Leukaemia  Skin | Low / research  High | chr7 | 124,812,385 | 124,939,825 |
| *PRSS1* | Pancreas | Medium / Low w high frequency | chr7 | 142,739,471 | 142,763,072 |
| *PTCH1* | CNS, Paediatric hereditary | Low / research | chr9 | 95,432,979 | 95,519,266 |
| *PTEN* | Breast, Digestive tract  CNS, Endocrin, Ovary, Paediatric hereditary | High  Low / research | chr10 | 87,853,624 | 87,981,930 |
| *PTPN11* | Leukaemia | Low / research | chr12 | 112,408,946 | 112,519,918 |
| *RAD50* | Breast, Ovary, Pancreas | Low / research | chr5 | 132,546,976 | 132,656,349 |
| *RAD51B* | Breast, Ovary, Pancreas | Low / research | chr14 | 67,855,031 | 68,693,118 |
| *RAD51C* | Breast, CNS, Pancreas, Prostate  Ovary | Low / research  High | chr17 | 58,682,601 | 58,745,611 |
| *RAD51D* | Breast, Pancreas, Prostate  Ovary | Low / research  High | chr17 | 35,082,220 | 35,129,860 |
| *RB1* | Paediatric hereditary | Low / research | chr13 | 48,293,750 | 48,491,890 |
| *RECQL4* | Paediatric hereditary | Low / research | chr8 | 144,501,287 | 144,527,833 |
| *RET* | CNS, Endocrin, Paediatric hereditary | Low / research | chr10 | 43,067,068 | 43,137,504 |
| *RINT1* | Breast | Low / research | chr7 | 105,522,201 | 105,577,677 |
| *RNF43* | Digestive tract | Medium / Low w high frequency | chr17 | 58,343,675 | 58,427,582 |
| *RPL5* | Leukaemia | Low / research | chr1 | 92,822,039 | 92,851,924 |
| *RPS20* | Digestive tract | Low / research | chr8 | 56,057,295 | 56,084,509 |
| *RTEL1* | Leukaemia | Low / research | chr20 | 63,647,809 | 63,706,253 |
| *RUNX1* | Leukaemia | Medium / Low w high frequency | chr21 | 34,777,800 | 34,898,690 |
| *SAMD9* | Leukaemia | Low / research | chr7 | 93,089,512 | 93,128,023 |
| *SAMD9L* | Leukaemia | Low / research | chr7 | 93,120,055 | 93,158,385 |
| *SBDS* | Leukaemia | Low / research | chr7 | 66,977,679 | 67,005,586 |
| *SDHA* | Kidney  Endocrin | Medium / Low w high frequency  Low / research | chr5 | 208,319 | 267,082 |
| *SDHAF2* | Endocrin | Low / research | chr11 | 61,420,123 | 61,456,733 |
| *SDHB* | Kidney  Endocrin | Medium / Low w high frequency  Low / research | chr1 | 17,008,721 | 17,064,032 |
| *SDHC* | Kidney  Endocrin | Medium / Low w high frequency  Low / research | chr1 | 161,304,380 | 161,373,206 |
| *SDHD* | Kidney  Endocrin | Medium / Low w high frequency  Low / research | chr11 | 112,076,872 | 112,105,794 |
| *SH2B3* | Leukaemia | Low / research | chr12 | 111,395,922 | 111,461,623 |
| *SLX4* | CNS | Low / research | chr16 | 3,571,180 | 3,621,606 |
| *SMAD4* | Digestive tract | High | chr18 | 51,019,613 | 51,095,045 |
| *SMARCA4* | CNS | Low / research | chr19 | 10,951,001 | 11,072,256 |
| *SMARCB1* | Paediatric hereditary | Low / research | chr22 | 23,776,931 | 23,844,540 |
| *SPINK1* | Pancreas | Medium / Low w high frequency | chr5 | 147,814,581 | 147,841,671 |
| *SRP72* | Leukaemia | Low / research | chr4 | 56,457,616 | 56,513,681 |
| *STIM1* | Leukaemia | Low / research | chr11 | 3,845,701 | 4,103,209 |
| *STK11* | Breast, Digestive tract  Ovary, Paediatric hereditary  Pancreas | High  Low / research  Medium / Low w high frequency | chr19 | 1,195,777 | 1,238,431 |
| *STN1* | Leukaemia | Low / research | chr10 | 103,846,828 | 103,928,249 |
| *SUFU* | Paediatric hereditary | Low / research | chr10 | 102,493,971 | 102,643,535 |
| *TERC* | Leukaemia | Medium / Low w high frequency | chr3 | 169,754,519 | 169,775,060 |
| *TERT* | Leukaemia  Skin | Medium / Low w high frequency  High | chr5 | 1,243,166 | 1,305,068 |
| *TINF2* | Leukaemia | Medium / Low w high frequency | chr14 | 24,229,642 | 24,252,623 |
| *TMEM127* | Endocrin | Low / research | chr2 | 96,238,514 | 96,275,997 |
| *TP53* | Breast  Ovary, Pancreas  CNS, Digestive tract, Endocrin, Leukaemia Paediatric hereditary | High  Medium / Low w high frequency  Low / research | chr17 | 7,658,420 | 7,697,490 |
| *TRIM37* | Kidney  Paediatric hereditary | Medium / Low w high frequency  Low / research | chr17 | 58,972,650 | 59,116,921 |
| *TSC1* | Kidney  CNS, Paediatric hereditary | High  Low / research | chr9 | 132,881,352 | 132,954,633 |
| *TSC2* | Kidney  CNS, Paediatric hereditary | High  Low / research | chr16 | 2,038,019 | 2,098,718 |
| *VHL* | Kidney  CNS, Endocrin | High  Low / research | chr3 | 10,131,777 | 10,163,667 |
| *WRAP53* | Leukaemia | Low / research | chr17 | 7,676,070 | 7,713,502 |
| *WRN* | Paediatric hereditary |  | chr8 | 31,023,809 | 31,186,138 |
| *WT1* | Kidney  Paediatric hereditary | Medium / Low w high frequency  Low / research | chr11 | 32,379,057 | 32,445,360 |
| *XPA* | Paediatric hereditary | Low / research | chr9 | 97,664,908 | 97,707,340 |
| *XPB/ERCC3* | Paediatric hereditary | Low / research | chr2 | 127,247,290 | 127,304,144 |
| *XPC* | Paediatric hereditary | Low / research | chr3 | 14,135,146 | 14,188,601 |
| *XPD/ERCC2* | Paediatric hereditary | Low / research | chr19 | 45,339,837 | 45,380,573 |
| *XPE/DDB1* | Paediatric hereditary | Low / research | chr11 | 61,289,455 | 61,343,807 |
| *XPF/ERCC4* | Paediatric hereditary | Low / research | chr16 | 13,910,154 | 13,962,348 |
| *XPG/ERCC5* | Paediatric hereditary | Low / research | chr13 | 102,836,032 | 102,885,995 |
| *XRCC2* | Breast | Low / research | chr7 | 152,634,776 | 152,686,141 |

CNS: Central Nervous System
