# Supplementary material for: Nanopore adaptive sampling accurately detects nucleotide variants and improves the characterization of large‐scale rearrangement for the diagnosis of cancer predisposition
Source: Clin Transl Med. 2025 Jan 9;15(1):e70138. doi: 10.1002/ctm2.70138 (PMC11714230; doi:10.1002/ctm2.70138)
Supplement: Supplementary file 7 — Supporting Information [file CTM2-15-e70138-s006.docx]

Supplementary Table S2: List of the 6 nucleotide variations detected by Nanopore sequencing and selected for confirmation by Sanger sequencing

| Gene | Mutations observed | | Patient | Coverage | Quality score |
| --- | --- | --- | --- | --- | --- |
| *GPT* | c.703_704delinsAA | p.(Arg235Asn) | #28 | 34 | 26 |
| *CFAP126* | c.27+1G>T | *Splicing* | #30 | 12 | 25 |
| *MSH3* | c.162_179del | p.(Ala57_Ala62del) | #8, #10 | 37 | 21 |
| *ANKRD26* | c.1868G>T | p.(Arg623Leu) | #6 | 23 | 23 |
| *TKFC* | c.703G>A | p.(Asp235Asn) | #29 | 16 | 25 |
| *BLM* | c.3341T>C | p.(Leu1114Pro) | #28 | 28 | 25 |
