# Supplementary material for: Nanopore adaptive sampling accurately detects nucleotide variants and improves the characterization of large‐scale rearrangement for the diagnosis of cancer predisposition
Source: Clin Transl Med. 2025 Jan 9;15(1):e70138. doi: 10.1002/ctm2.70138 (PMC11714230; doi:10.1002/ctm2.70138)
Supplement: Supplementary file 8 — Supporting Information [file CTM2-15-e70138-s002.docx]

**Supplementary methods**

*DNA extraction.*

DNA was extracted with the Monarch Genomic DNA purification kit (New England Biolabs) according to the manufacturer's protocol, and quantified using a fluorimetric assay on a Qubit device 4 (Life Technologies; Thermo Fisher Scientific, Inc.).

*Sanger sequencing*

Twenty nanograms of gDNA were amplified by PCR with specific forward and reverse primers (ANKRD26-F: TTGAGATGGGGGAAATGTTGGAA, -R: AGCTTTGGGAGACTACATCATTC ; BLM-F: TGGTCTGGAAATGGGTTATGATGA, -R: ACATTCAAAACAGATGACTTACCCA ; CFAP126-F: GCAGCCTCTGCTCATTGGTA, -R: ATACCTTTGGATCGCCCCTG ; GPT-F: GCCCTTCACTCACTGTCAACT, -R: CACACAGTCCAGGGCGA ; MSH3-F: AGCGGTTTTGAGCCGATTCT, -R: TCCCACCTTCCCCTTCTTCA ; TFKC-F: CCCAGATGCAGCTCATTCCT, -R: CATGACACCCCAGGTGAGAC) and AmpliTaq Gold Fast PCR Master Mix (Applied Biosystems) by following manufacturer’s instructions. Then, PCR products were purified with PCR ExoSAP-IT (Applied Biosystems) by following manufacturer’s instructions. Two µl of purified PCR products were labeled with the BigDye Terminator v3.1 Cycle Sequencing kit (Applied Biosystems), and then purified with BigDye XTerminator Purification Kit (Applied Biosystems). Finally, final products were sequenced on a 3500 genetic analyzer (Applied Biosystems). Data were visualized with Alamut software (Sophia Genetics).

**Supplementary Figures**

5824 samples with known alterations detected by NGS and/or MLPA from 2017 to 2022

4717 samples with known alterations detected by NGS and/or MLPA from 2017 to 2022

1107 samples without available consents

11 samples with *BRCA1* alterations

6 samples with *BRCA2* alterations

3 samples with *PALB2* alterations

6 samples with *MLH1* alterations

4 samples without alterations

6 samples with MLPA confirmed LSR

5 samples with NGS detected SNV

2 samples with MLPA confirmed LSR

4 samples with NGS detected SNV

3 samples with NGS detected SNV

1 sample with MLPA confirmed LSR

5 samples with NGS detected SNV

- 20 samples analyzed once by Nanopore Adaptive sampling sequencing
- 6 samples analyzed twice by Nanopore Adaptive sampling sequencing
- 4 samples analyzed three times by Nanopore Adaptive sampling sequencing

Data analysis with SeqOne Genomics pipeline

Selection of 6 new variants representative of different mutation types

Sanger sequencing

**Supplemental_Fig_S1: Consort plot of the design and the course of the study.** We selected samples with representative alterations that can be observed in a routine activity with technics used in a routine molecular diagnosis lab. At the end of the study, we selected variants by applying thresholds on coverage and sequencing quality. Six variants in 6 different genes were selected for a Sanger sequencing analysis. These variants represented different types of alterations (small deletion, transition, transversion, double point mutations).

A

B

C

E

D

G

F

**Supplemental_Fig_S2: Technical set up of adaptive sampling. A.** Contrary to the amount of library in fmoles, the library amount in nanograms (ng) did not influence sequencing throughput. **B-D.** The sequencing throughput had no influence neither on percentage of reads passing filters (**B**), nor on percentage of reads in target (**C**), nor on enrichment of targets (**D**). **E.** Enrichment is directly linked to the percentage of reads in target. **F**. Enrichment, translating the capacity of pores to select target sequences, directly influenced the coverage of target genes. Indeed, the higher the enrichment, the higher the coverage. **G**. For each run were plotted the coverage obtained for the shallow Whole Genome Sequencing (rejected reads), the coverage obtained on the manifest for the same run, and the enrichment calculated by dividing the coverage on the manifest by the coverage of the rejected reads.

**
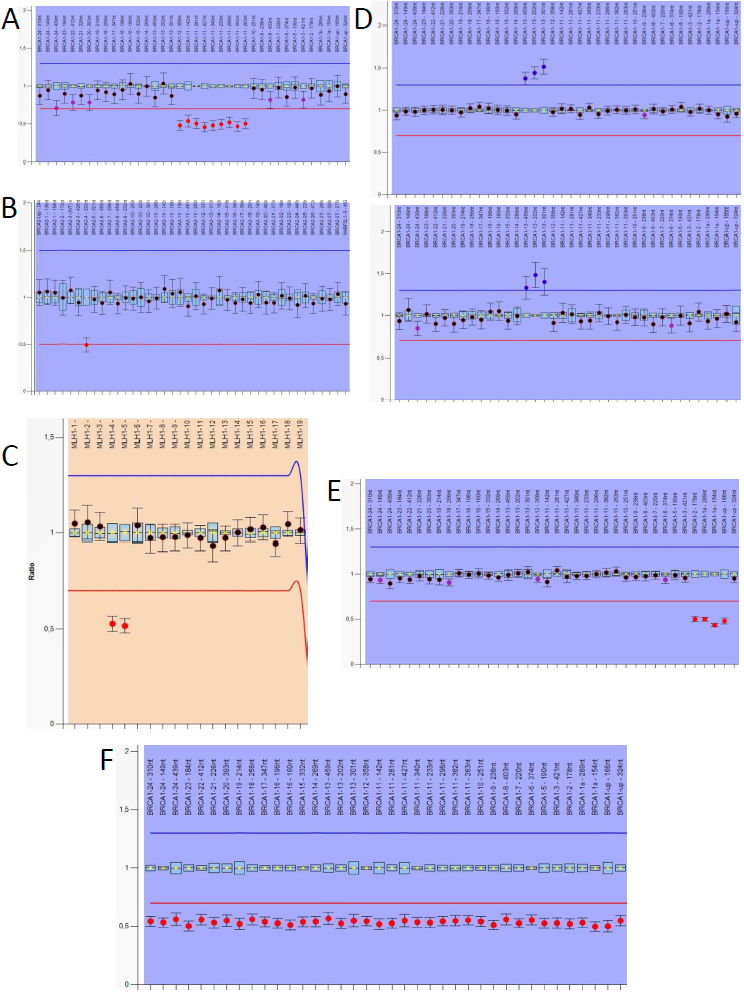
**

**Supplemental_Fig_S3: Coffalyser (software from MRC Holland) visualization of large scale rearrangements (LSR) obtained by MLPA.** **A.** Sample #5 had a deletion of exons 11 and 12 of *BRCA1* gene. **B.** Sample #22 had a deletion of exon 4 of *BRCA2* gene. **C.** Sample #17 harbored a deletion of exons 4 and 5 of *MLH1* gene. **D.** Samples #9 (upper) and #10 (lower) were relatives who had both an exon 13 duplication in *BRCA1* gene. **E.** Sample #7 had a deletion of a part of the promoter, exon 1a and exon 2 of *BRCA1* gene. **F.** Sample #8 presented a complete deletion of *BRCA1*.

**
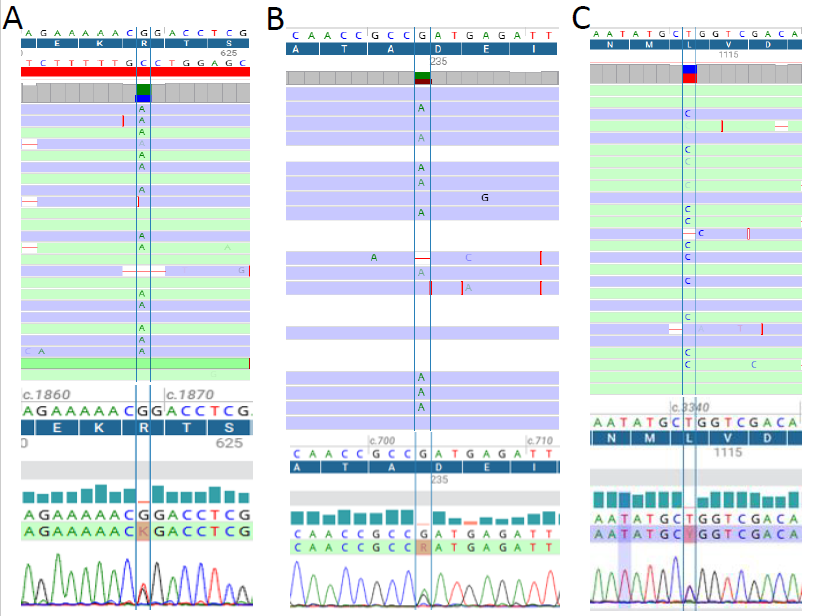
**

**Supplemental_Fig_S4: Confirmation by Sanger sequencing of variants detected with ONT sequencing.** We confirmed the mutation c.1868G>T in the *ANKRD26* gene (**A**), the mutation c.703G>A in the *TKFC* gene (**B**), and the mutation c.3341T>C in the *BLM* gene (**C**).


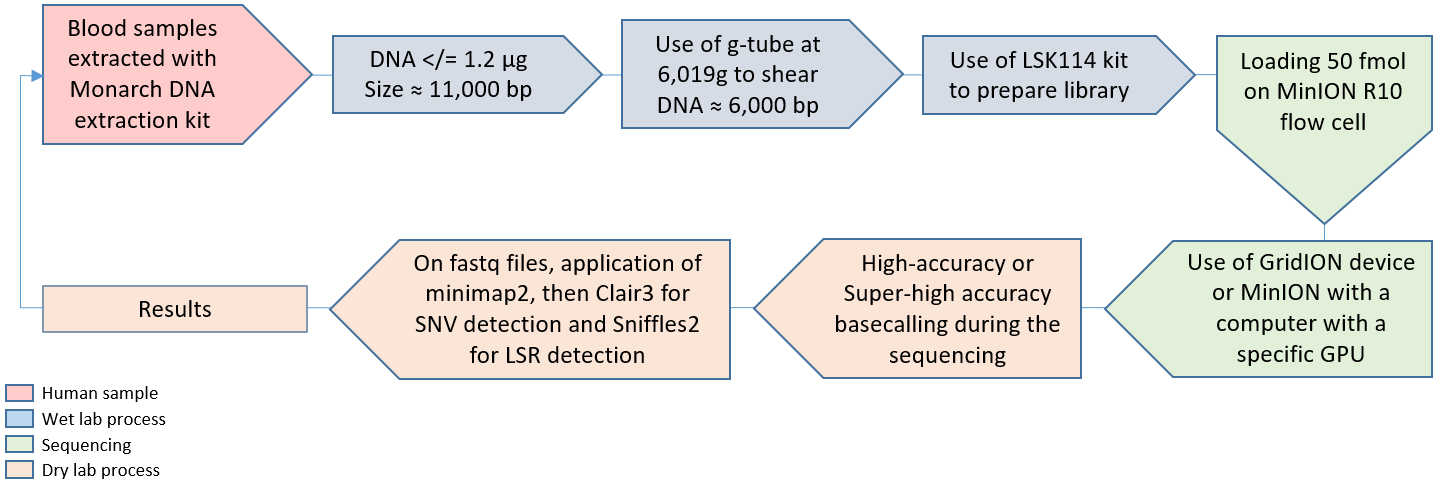


**Supplemental_Fig_S5: Graphical flowchart showing the workflow for adaptive sampling analysis.** After the extraction of DNA from blood samples with Monarch DNA extraction kit, we sheared a high 1.2 µg of less high quality DNA using g-tube at 6,019g. Then, libraries were prepared with LSK114 and load on MinION flow cells. During the adaptive sampling sequencing, high or super-high accuracy basecalling was performed to obtain fastq files. Finally, results were obtained after bioinformatics analysis using minimap2, Clair3 for SNV and Sniffles2 for LSR.

Supplementary Table S1: Detail of genes present in the adaptive sampling bed file

| Gene | Related disease | Level of risk | chromosome | Start | stop |
| --- | --- | --- | --- | --- | --- |
| *ABRAXAS1* | Breast | Low / research | chr4 | 83,449,517 | 83,495,100 |
| *ANKRD26* | Leukaemia | Medium / Low w high frequency | chr10 | 26,994,116 | 27,110,494 |
| *ACD* | Leukaemia | Low / research | chr16 | 67,647,511 | 67,670,260 |
| *APC* | Digestive tract  CNS, Paediatric hereditary, Pancreas | High  Low / research | chr5 | 112,727,884 | 112,856,239 |
| *ATG2B* | Leukaemia | Low / research | chr14 | 96,269,194 | 96,373,341 |
| *ATM* | Breast, Digestive tract, Pancreas, Prostate  Ovary, Paediatric hereditary | Medium / Low w high frequency  Low / research | chr11 | 108,213,066 | 108,379,102 |
| *ATR* | Breast, Leukaemia, Ovary | Low / research | chr3 | 142,439,234 | 142,588,733 |
| *AXIN2* | Digestive tract | Medium / Low w high frequency | chr17 | 65,518,562 | 65,571,648 |
| *BAP1* | Kidney  Skin | Medium / Low w high frequency  High | chr3 | 52,391,007 | 52,420,008 |
| *BARD1* | Breast, Ovary, Prostate | Low / research | chr2 | 214,715,645 | 214,819,683 |
| *BLM* | Digestive tract, Leukaemia  Paediatric hereditary | Medium / Low w high frequency  Low / research | chr15 | 90,707,345 | 90,826,166 |
| *BMPR1A* | Digestive tract | High | chr10 | 86,746,618 | 86,937,969 |
| *BRCA1* | Breast, Ovary, Prostate  Pancreas  Skin | High  Medium / Low w high frequency  Low / research | chr17 | 43,034,294 | 43,135,364 |
| *BRCA2* | Breast, Ovary, Prostate  Pancreas  CNS, Skin | High  Medium / Low w high frequency  Low / research | chr13 | 32,305,085 | 32,410,268 |
| *BRIP1* | Breast, CNS, Ovary, Pancreas, Prostate | Low / research | chr17 | 61,669,138 | 61,873,528 |
| *CDH1* | Breast  Digestive tract  Ovary | High  Medium / Low w high frequency  Low / research | chr16 | 68,727,291 | 68,845,537 |
| *CDK4* | Skin | High | chr12 | 57,737,726 | 57,762,310 |
| *CDKN1B* | CNS | Low / research | chr12 | 12,675,497 | 12,732,325 |
| *CDKN1C* | Kidney | Medium / Low w high frequency | chr11 | 2,873,212 | 2,895,771 |
| *CDKN2A* | Pancreas  Skin | Medium / Low w high frequency  High | chr9 | 21,957,751 | 22,004,392 |
| *CDKN2B* | Skin | High | chr9 | 21,992,902 | 22,019,305 |
| *CEBPA* | Leukaemia | Medium / Low w high frequency | chr19 | 33,289,933 | 33,312,534 |
| *CFTR* | Pancreas | Medium / Low w high frequency | chr7 | 117,470,025 | 117,678,665 |
| *CHEK2* | Breast, Prostate  Digestive tract, Endocrin, Ovary, Pancreas, Prostate | Medium / Low w high frequency  Low / research | chr22 | 28,677,742 | 28,751,820 |
| *CREBBP* | Leukaemia, Paediatric hereditary | Low / research | chr16 | 3,715,053 | 3,890,713 |
| *CTC1* | Leukaemia | Low / research | chr17 | 8,214,814 | 8,258,056 |
| *CTNNA1* | Digestive tract | Low / research | chr5 | 138,743,425 | 138,945,034 |
| *DDX41* | Leukaemia | Medium / Low w high frequency | chr5 | 177,501,576 | 177,526,961 |
| *DICER1* | Endocrin, Paediatric hereditary | Low / research | chr14 | 95,076,243 | 95,168,010 |
| *DKC1* | Leukaemia | Low / research | chrX | 154,752,863 | 154,787,689 |
| *DNAH9* | Leukaemia | Low / research | chr17 | 11,588,469 | 11,979,748 |
| *DNAJC21* | Leukaemia | Low / research | chr5 | 34,919,558 | 34,968,964 |
| *ELANE* | Leukaemia | Low / research | chr19 | 841,013 | 866,247 |
| *EPCAM* | Breast  Digestive tract, Prostate  Ovary, Pancreas | Low / research  High  Medium / Low w high frequency | chr2 | 47,359,310 | 47,397,020 |
| *ERCC6L2* | Leukaemia | Low / research | chr9 | 95,865,690 | 96,028,447 |
| *ETV6* | Leukaemia | Medium / Low w high frequency | chr12 | 11,639,673 | 11,905,377 |
| *EVI1/MECOM* | Leukaemia | Low / research | chr3 | 169,073,507 | 169,673,712 |
| *EXT1* | Paediatric hereditary | Low / research | chr8 | 117,784,489 | 118,121,826 |
| *EXT2* | Paediatric hereditary | Low / research | chr11 | 44,085,677 | 44,261,962 |
| *FAN1* | Digestive tract | Low / research | chr15 | 30,893,864 | 30,953,085 |
| *FANCA* | CNS, Leukaemia, Paediatric hereditary | Low / research | chr16 | 89,727,548 | 89,826,647 |
| *FANCB* | CNS, Leukaemia, Paediatric hereditary | Low / research | chrX | 14,830,183 | 14,883,064 |
| *FANCC* | CNS, Leukaemia, Paediatric hereditary, Pancreas | Low / research | chr9 | 95,089,053 | 95,327,709 |
| *FANCD2* | CNS, Leukaemia, Paediatric hereditary | Low / research | chr3 | 10,016,436 | 10,111,932 |
| *FANCE* | CNS, Leukaemia | Low / research | chr6 | 35,442,337 | 35,477,102 |
| *FANCF* | CNS, Leukaemia, Paediatric hereditary | Low / research | chr11 | 22,612,532 | 22,635,823 |
| *FANCG* | CNS, Leukaemia, Paediatric hereditary, Pancreas | Low / research | chr9 | 35,063,855 | 35,089,969 |
| *FANCI* | CNS, Leukaemia | Low / research | chr15 | 89,233,978 | 89,327,131 |
| *FANCL* | CNS | Low / research | chr2 | 58,149,242 | 58,251,345 |
| *FANCM* | CNS | Low / research | chr14 | 45,125,929 | 45,210,890 |
| *FH* | Kidney  Endocrin | High  Low / research | chr1 | 241,487,602 | 241,529,755 |
| *FLCN* | Digestive tract  Kidney | Low / research  High | chr17 | 17,202,211 | 17,247,168 |
| *GALNT12* | Digestive tract | Low / research | chr9 | 98,797,670 | 98,860,081 |
| *GATA2* | Leukaemia | Medium / Low w high frequency | chr3 | 128,469,426 | 128,503,201 |
| *GPC2* | Kidney | Medium / Low w high frequency | chr7 | 100,159,605 | 100,187,381 |
| *GREM1* | Digestive tract | Medium / Low w high frequency | chr15 | 32,708,003 | 32,755,106 |
| *GSKIP* | Leukaemia | Low / research | chr14 | 96,353,451 | 96,397,286 |
| *HOXB13* | Breast  Prostate | Low / research  High | chr17 | 48,714,762 | 48,738,750 |
| *HRAS* | Paediatric hereditary | Low / research | chr11 | 522,241 | 545,576 |
| *IGF2* | Endocrin | Low / research | chr11 | 2,119,111 | 2,148,974 |
| *KRAS* | Leukaemia, Paediatric hereditary | Low / research | chr12 | 25,195,245 | 25,260,929 |
| *LIG4* | Leukaemia | Low / research | chr13 | 108,197,438 | 108,228,368 |
| *MAX* | Endocrin | Low / research | chr14 | 65,065,126 | 65,112,517 |
| *MBD4* | Leukaemia | Low / research | chr3 | 129,420,949 | 129,450,009 |
| *MC1R* | Skin | Medium / Low w high frequency | chr16 | 89,904,846 | 89,930,951 |
| *MEN1* | CNS, Endocrin, Paediatric hereditary | Low / research | chr11 | 64,793,515 | 64,820,686 |
| *MET* | Kidney | High | chr7 | 116,662,195 | 116,808,377 |
| *MITF* | Skin | Medium / Low w high frequency | chr3 | 69,729,464 | 69,978,332 |
| *MLH1* | Breast, CNS  Digestive tract, Prostate  Ovary, Pancreas | Low / research  High  Medium / Low w high frequency | chr3 | 36,983,517 | 37,060,846 |
| *MLH3* | Digestive tract  Prostate | Medium / Low w high frequency  High | chr14 | 75,003,774 | 75,061,467 |
| *MRE11* | Ovary, Pancreas, Prostate | Low / research | chr11 | 94,405,569 | 94,503,844 |
| *MSH2* | Breast, CNS  Digestive tract, Prostate  Ovary, Pancreas | Low / research  High  Medium / Low w high frequency | chr2 | 47,393,118 | 47,673,146 |
| *MSH3* | Digestive tract | Medium / Low w high frequency | chr5 | 80,644,651 | 80,886,815 |
| *MSH6* | Breast, CNS  Digestive tract, Prostate  Ovary, Pancreas | Low / research  High  Medium / Low w high frequency | chr2 | 47,773,144 | 47,816,953 |
| *MUTYH* | Digestive tract | High | chr1 | 45,319,241 | 45,350,115 |
| *NAF1* | Leukaemia | Low / research | chr4 | 163,118,668 | 163,176,890 |
| *NAPRT* | Leukaemia | Low / research | chr8 | 143,564,784 | 143,588,330 |
| *NBN* | Breast, Leukaemia, Ovary, Pancreas, Prostate | Low / research | chr8 | 89,914,514 | 89,994,682 |
| *NBR1* | Kidney | Low / research | chr17 | 43,160,481 | 43,221,689 |
| *NBR2* | Breast, Kidney | Low / research | chr17 | 43,115,551 | 43,163,649 |
| *NF1* | Breast, CNS, Endocrin, Paediatric hereditary | Low / research | chr17 | 31,084,976 | 31,387,675 |
| *NF2* | CNS, Paediatric hereditary | Low / research | chr22 | 29,593,632 | 29,708,598 |
| *NOP10* | Leukaemia | Low / research | chr15 | 34,331,719 | 34,353,136 |
| *NHP2* | Leukaemia | Low / research | chr5 | 178,139,463 | 178,163,885 |
| *NRAS* | Leukaemia, Paediatric hereditary | Low / research | chr1 | 114,694,468 | 114,726,771 |
| *NSD1* | CNS, Endocrin, Paediatric hereditary | Low / research | chr5 | 177,123,772 | 177,310,213 |
| *NTHL1* | Digestive tract | Medium / Low w high frequency | chr16 | 2,029,814 | 2,057,866 |
| *PALB2* | Breast  CNS, Prostate  Ovary, Pancreas | High  Low / research  Medium / Low w high frequency | chr16 | 23,593,164 | 23,651,310 |
| *PARN* | Leukaemia | Low / research | chr16 | 14,425,700 | 14,640,260 |
| *PAX5* | Leukaemia | Low / research | chr9 | 36,823,268 | 37,044,268 |
| *PHOX2B* | CNS, Endocrin, Paediatric hereditary | Low / research | chr4 | 41,734,081 | 41,758,725 |
| *PMS2* | Breast, CNS  Digestive tract, Prostate  Ovary, Pancreas | Low / research  High  Medium / Low w high frequency | chr7 | 5,960,924 | 6,019,049 |
| *POLD1* | Digestive tract | Medium / Low w high frequency | chr19 | 50,374,322 | 50,428,018 |
| *POLE* | Digestive tract | Medium / Low w high frequency | chr12 | 132,613,761 | 132,697,342 |
| *POLH* | Paediatric hereditary | Low / research | chr6 | 43,566,184 | 43,630,523 |
| *POT1* | Leukaemia  Skin | Low / research  High | chr7 | 124,812,385 | 124,939,825 |
| *PRSS1* | Pancreas | Medium / Low w high frequency | chr7 | 142,739,471 | 142,763,072 |
| *PTCH1* | CNS, Paediatric hereditary | Low / research | chr9 | 95,432,979 | 95,519,266 |
| *PTEN* | Breast, Digestive tract  CNS, Endocrin, Ovary, Paediatric hereditary | High  Low / research | chr10 | 87,853,624 | 87,981,930 |
| *PTPN11* | Leukaemia | Low / research | chr12 | 112,408,946 | 112,519,918 |
| *RAD50* | Breast, Ovary, Pancreas | Low / research | chr5 | 132,546,976 | 132,656,349 |
| *RAD51B* | Breast, Ovary, Pancreas | Low / research | chr14 | 67,855,031 | 68,693,118 |
| *RAD51C* | Breast, CNS, Pancreas, Prostate  Ovary | Low / research  High | chr17 | 58,682,601 | 58,745,611 |
| *RAD51D* | Breast, Pancreas, Prostate  Ovary | Low / research  High | chr17 | 35,082,220 | 35,129,860 |
| *RB1* | Paediatric hereditary | Low / research | chr13 | 48,293,750 | 48,491,890 |
| *RECQL4* | Paediatric hereditary | Low / research | chr8 | 144,501,287 | 144,527,833 |
| *RET* | CNS, Endocrin, Paediatric hereditary | Low / research | chr10 | 43,067,068 | 43,137,504 |
| *RINT1* | Breast | Low / research | chr7 | 105,522,201 | 105,577,677 |
| *RNF43* | Digestive tract | Medium / Low w high frequency | chr17 | 58,343,675 | 58,427,582 |
| *RPL5* | Leukaemia | Low / research | chr1 | 92,822,039 | 92,851,924 |
| *RPS20* | Digestive tract | Low / research | chr8 | 56,057,295 | 56,084,509 |
| *RTEL1* | Leukaemia | Low / research | chr20 | 63,647,809 | 63,706,253 |
| *RUNX1* | Leukaemia | Medium / Low w high frequency | chr21 | 34,777,800 | 34,898,690 |
| *SAMD9* | Leukaemia | Low / research | chr7 | 93,089,512 | 93,128,023 |
| *SAMD9L* | Leukaemia | Low / research | chr7 | 93,120,055 | 93,158,385 |
| *SBDS* | Leukaemia | Low / research | chr7 | 66,977,679 | 67,005,586 |
| *SDHA* | Kidney  Endocrin | Medium / Low w high frequency  Low / research | chr5 | 208,319 | 267,082 |
| *SDHAF2* | Endocrin | Low / research | chr11 | 61,420,123 | 61,456,733 |
| *SDHB* | Kidney  Endocrin | Medium / Low w high frequency  Low / research | chr1 | 17,008,721 | 17,064,032 |
| *SDHC* | Kidney  Endocrin | Medium / Low w high frequency  Low / research | chr1 | 161,304,380 | 161,373,206 |
| *SDHD* | Kidney  Endocrin | Medium / Low w high frequency  Low / research | chr11 | 112,076,872 | 112,105,794 |
| *SH2B3* | Leukaemia | Low / research | chr12 | 111,395,922 | 111,461,623 |
| *SLX4* | CNS | Low / research | chr16 | 3,571,180 | 3,621,606 |
| *SMAD4* | Digestive tract | High | chr18 | 51,019,613 | 51,095,045 |
| *SMARCA4* | CNS | Low / research | chr19 | 10,951,001 | 11,072,256 |
| *SMARCB1* | Paediatric hereditary | Low / research | chr22 | 23,776,931 | 23,844,540 |
| *SPINK1* | Pancreas | Medium / Low w high frequency | chr5 | 147,814,581 | 147,841,671 |
| *SRP72* | Leukaemia | Low / research | chr4 | 56,457,616 | 56,513,681 |
| *STIM1* | Leukaemia | Low / research | chr11 | 3,845,701 | 4,103,209 |
| *STK11* | Breast, Digestive tract  Ovary, Paediatric hereditary  Pancreas | High  Low / research  Medium / Low w high frequency | chr19 | 1,195,777 | 1,238,431 |
| *STN1* | Leukaemia | Low / research | chr10 | 103,846,828 | 103,928,249 |
| *SUFU* | Paediatric hereditary | Low / research | chr10 | 102,493,971 | 102,643,535 |
| *TERC* | Leukaemia | Medium / Low w high frequency | chr3 | 169,754,519 | 169,775,060 |
| *TERT* | Leukaemia  Skin | Medium / Low w high frequency  High | chr5 | 1,243,166 | 1,305,068 |
| *TINF2* | Leukaemia | Medium / Low w high frequency | chr14 | 24,229,642 | 24,252,623 |
| *TMEM127* | Endocrin | Low / research | chr2 | 96,238,514 | 96,275,997 |
| *TP53* | Breast  Ovary, Pancreas  CNS, Digestive tract, Endocrin, Leukaemia Paediatric hereditary | High  Medium / Low w high frequency  Low / research | chr17 | 7,658,420 | 7,697,490 |
| *TRIM37* | Kidney  Paediatric hereditary | Medium / Low w high frequency  Low / research | chr17 | 58,972,650 | 59,116,921 |
| *TSC1* | Kidney  CNS, Paediatric hereditary | High  Low / research | chr9 | 132,881,352 | 132,954,633 |
| *TSC2* | Kidney  CNS, Paediatric hereditary | High  Low / research | chr16 | 2,038,019 | 2,098,718 |
| *VHL* | Kidney  CNS, Endocrin | High  Low / research | chr3 | 10,131,777 | 10,163,667 |
| *WRAP53* | Leukaemia | Low / research | chr17 | 7,676,070 | 7,713,502 |
| *WRN* | Paediatric hereditary |  | chr8 | 31,023,809 | 31,186,138 |
| *WT1* | Kidney  Paediatric hereditary | Medium / Low w high frequency  Low / research | chr11 | 32,379,057 | 32,445,360 |
| *XPA* | Paediatric hereditary | Low / research | chr9 | 97,664,908 | 97,707,340 |
| *XPB/ERCC3* | Paediatric hereditary | Low / research | chr2 | 127,247,290 | 127,304,144 |
| *XPC* | Paediatric hereditary | Low / research | chr3 | 14,135,146 | 14,188,601 |
| *XPD/ERCC2* | Paediatric hereditary | Low / research | chr19 | 45,339,837 | 45,380,573 |
| *XPE/DDB1* | Paediatric hereditary | Low / research | chr11 | 61,289,455 | 61,343,807 |
| *XPF/ERCC4* | Paediatric hereditary | Low / research | chr16 | 13,910,154 | 13,962,348 |
| *XPG/ERCC5* | Paediatric hereditary | Low / research | chr13 | 102,836,032 | 102,885,995 |
| *XRCC2* | Breast | Low / research | chr7 | 152,634,776 | 152,686,141 |

CNS: Central Nervous System

Supplementary Table S2: List of the 6 nucleotide variations detected by Nanopore sequencing and selected for confirmation by Sanger sequencing

| Gene | Mutations observed | | Patient | Coverage | Quality score |
| --- | --- | --- | --- | --- | --- |
| *GPT* | c.703_704delinsAA | p.(Arg235Asn) | #28 | 34 | 26 |
| *CFAP126* | c.27+1G>T | *Splicing* | #30 | 12 | 25 |
| *MSH3* | c.162_179del | p.(Ala57_Ala62del) | #8, #10 | 37 | 21 |
| *ANKRD26* | c.1868G>T | p.(Arg623Leu) | #6 | 23 | 23 |
| *TKFC* | c.703G>A | p.(Asp235Asn) | #29 | 16 | 25 |
| *BLM* | c.3341T>C | p.(Leu1114Pro) | #28 | 28 | 25 |
